# DataXflow: Synergizing data-driven modeling with best parameter fit and optimal control – An efficient data analysis for cancer research

**DOI:** 10.1016/j.csbj.2024.04.010

**Published:** 2024-04-08

**Authors:** Samantha A.W. Crouch, Jan Krause, Thomas Dandekar, Tim Breitenbach

**Affiliations:** Department of Bioinformatics, Biocenter, University of Würzburg, Am Hubland 97074, Würzburg, Germany

**Keywords:** Data-driven modeling, Best parameter fit, Optimal control, Systematic drug target identification, Most effective network influence, External stimuli, Optimal drug combination, Identifying synergistic drugs

## Abstract

Building data-driven models is an effective strategy for information extraction from empirical data. Adapting model parameters specifically to data with a best fitting approach encodes the relevant information into a mathematical model. Subsequently, an optimal control framework extracts the most efficient targets to steer the model into desired changes via external stimuli. The DataXflow software framework integrates three software pipelines, D2D for model fitting, a framework solving optimal control problems including external stimuli and JimenaE providing graphical user interfaces to employ the other frameworks lowering the barriers for the need of programming skills, and simultaneously automating reoccurring modeling tasks. Such tasks include equation generation from a graph and script generation allowing also to approach systems with many agents, like complex gene regulatory networks. A desired state of the model is defined, and therapeutic interventions are modeled as external stimuli. The optimal control framework purposefully exploits the model-encoded information by providing those external stimuli that effect the desired changes most efficiently. The implementation of DataXflow is available under https://github.com/MarvelousHopefull/DataXflow.

We showcase its application by detecting specific drug targets for a therapy of lung cancer from measurement data to lower proliferation and increase apoptosis. By an iterative modeling process refining the topology of the model, the regulatory network of the tumor is generated from the data. An application of the optimal control framework in our example reveals the inhibition of AURKA and the activation of CDH1 as the most efficient drug target combination. DataXflow paves the way to an agile interplay between data generation and its analysis potentially accelerating cancer research by an efficient drug target identification, even in complex networks.

## Graphical Abstract

**Figure:**
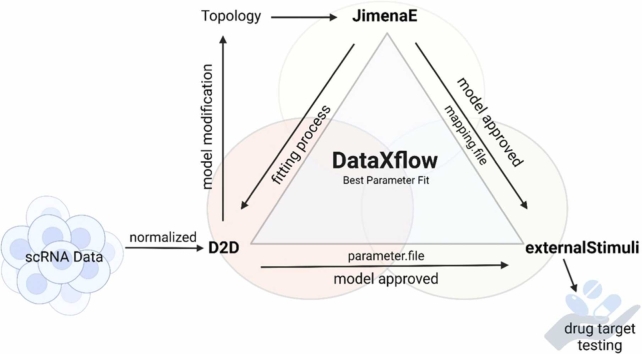

## 1 Introduction

One benefit of modeling biological processes with differential equations is to test our understanding of the real process quantitatively. Such a test validates the hypotheses generated with this differential model against the data measured in real experiments. Moreover, differential equations allow to quantify predictions and compare it with real data from the experiment.

The quantification of deviations even allows to focus on these parts of a model with the biggest potential of improvement, which is having the biggest deviation from the data, and to include relevant effects purposefully. Once a model fits sufficiently well to measured data, which may mean it cannot be rejected based on the data as an explanation for the processes generating this data, one interesting question is how to influence the investigated biological system to steer it to a desired state. Such an influence could be a therapy turning a pathological state into a physiological one implemented as, e.g., drugs modeled by external stimuli. If there are many potential choices, one would like to obtain the best working one. Depending on the accuracy of the model, a time-saving preselection of potential candidates can already be derived from the model with a suitable framework.

A mathematical model, which describes relations in a system, usually contains topological information. By the term topology, we mean what entity directly influences which one as well as if and how they interact, like an interaction activates or inhibits the affected entity as genes in a gene regulatory network regulating each other (Fig. 2). Once the topology of a system is fixed, the question is which functions to choose to model the interactions in a continuous manner to quantify the strength of interactions, causes and their effects. Such a function is determined up to parameters that need to be fitted to adapt the model output best to the data recorded. In other words, the parameters of the functions quantify the interaction strengths of the relations of the entities encoded in the topology.

There are already some mathematical frameworks developed for fitting parameters of a model to the data [16], [17], [21], [27], [46], [47], [51], [54]. With these frameworks one can figure out a mathematical model that fits the data best, which may be supported by a decision based on a statistical test if the model cannot be rejected based on the data. This means that the deviations between model and data are likely only caused by noise in the data and not a systematic model error missing important relations.

The task to select the best external stimuli to influence a system in a desired way, can be formulated as an optimal control problem. There are other approaches utilizing techniques from optimization and optimal control to calculate optimal dosages of drugs for therapies [10] and the strength of a treatment, like radiation [18], [19] and thus optimize therapies. Furthermore, there are simulation-based approaches to optimize dosing schedules [12], [13], [42] and techniques from game theory [59] to optimize therapies. Our approach is different since it aims at systematically identifying the most efficient intervention points in a network to steer the network to a desired target state by equipping the corresponding optimal control problem with an $L^{1}$-cost term of the controls [53]. This $L^{1}$-cost forces the time curve of optimal controls to constant zero in case their effect with respect to steering the network into the desired state is inefficient. The controls act as the external stimuli, like drugs. If they have been ruled out as inefficient, they are modeled by the corresponding control being a constant zero function. All controls that have not been ruled out are a representative of a drug target and efficient to steer the system according to our desire.

To solve such problems, tailored MATLAB scripts have been developed [6], [8] utilizing corresponding solution algorithms. The overall intention of this approach is to identify promising drug targets and drug combinations from measurement data to design promising follow up experiments to guide experimental lab work where in the best case these suggestions are validated successfully avoiding a resource consuming uninformed trial-and-error search by lab experiments. We remark that our provided software pipeline can be used for dosage calculations as well without any change if the underlying model with the external stimuli are fit accordingly. Implementing time curves as a drug administration is discussed in [9][Discussion 4.3] extending the model with respect to the distribution/metabolism of the drugs with further dynamics.

**Fig. 1:**
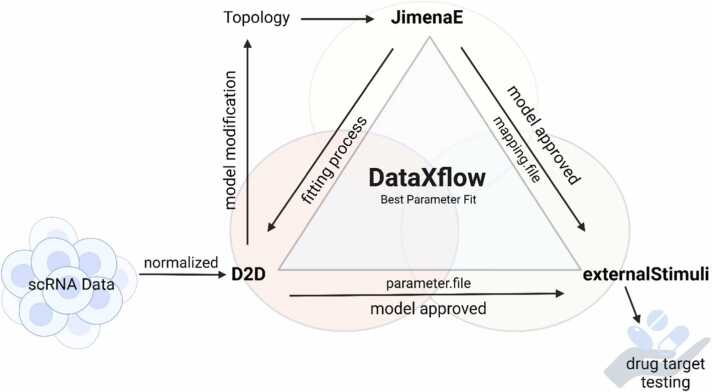
**DataXflow.** All three main steps, including JimenaE, D2D, and externalStimuli, are shown in the workflow. The input data consists of real data, such as single-cell data, which must undergo prior normalization. The output topology is crafted in yED. Subsequently, this topology is opened in JimenaE to generate scripts to use D2D, an integral part of JimenaE, for achieving the best parameter fit on the input data. An iterative topology adaption might be necessary to generate a fitting model. Once the model garners approval, the identification of drug targets using the external stimuli framework is done by generating necessary scripts automatically. To complete this process, the "mapping.file" from JimenaE and the "parameter.file" from D2D, generated post-fitting through "arExportPEtab," are essential. It's important to note that D2D and external stimuli require access to MATLAB. The normalization is conducted using the provided Python script, and all relevant scripts are accessible in the git repository under https://github.com/MarvelousHopefull/DataXflow. Created with BioRender.com.

The different tasks required for identifying optimal drug targets from data can be solved with different tools, each requiring different scripts to start the underlying solution routines in their developed environment. Consequently, the aim of this work is to provide a software tool that unifies the usage of several software tools all needed for the end-to-end process consisting of finding a well-fitting model and analyzing the best-working external stimuli to obtain a desired outcome of an experiment. We choose the D2D framework [46], [47] for best parameter fitting and the external stimuli framework [6], [9], [8] to find the best influencing strategy via optimal control and external stimuli. The software suite JimenaE [24] is selected as the hub for the graphical user interaction to provide necessary information for the other two frameworks. Within the DataXflow pipeline, which describes an end-to-end process of extracting promising intervention points from measurement data to steer a system into a desired state, available under https://github.com/MarvelousHopefull/DataXflow, the mentioned components fulfill the following tasks. The D2D framework, based on MATLAB, fits a given model to data. The external stimuli framework, based on MATLAB, determines efficient intervention points given a model, which is best fit by D2D. JimenaE, based on Java, has the task to provide graphical user interfaces (GUIs) to read in model topologies and to automatically derive the model equations according to the SQUAD model [31]. The SQUAD model is a system of coupled ordinary differential equations (ODEs) describing the regulation of genes from a graph representing the model topology. JimenaE sets up the corresponding scripts with all specific information via GUIs to run the other two components without any specific MATLAB programming skills. Our current implementation of the presented framework is illustrated in Fig. 1.

We aim at providing a software tool for end-to-end data-driven modeling to identify efficient drug targets given experimental data to a broad audience, even outside the data science/bioinformatics community, by automating and integrating a lot of tedious or data-science specific tasks and thus also taking user-friendliness of our software framework into account.

The advantage, even for bioinformaticians, is to save a lot of copy work to generate the scripts manually. In addition, the automated model generation helps to reduce errors that happen noting down complex equations manually. Being able to integrate the whole data analysis in one tool provides overview and minimizes chances for human errors in the modeling process leading to unnecessary loss of time. Moreover, the automated model generation particularly shows its benefits if we modify the model topology only by a few interactions. These iterative changes may often appear when modeling a system from the scratch not having the full knowledge about the topology of the system one is modeling and testing which topology, resp., model could be the most plausible based on the available data. It is quite natural to test data-driven if an interaction between genes seems reasonable given the data. The test is done by inspecting if the fit is improved with the corresponding interaction in the topology. In a complex scenario with many interactions, having to do these changes manually would be very challenging.

An optimal control framework integrated into DataXflow exploits the encoded information purposefully and efficiently. Defining a desired state of the system, we model therapeutic interventions as external stimuli. The optimal control framework considers the influence of the external stimuli on the network holistically providing one way to automatically extract promising intervention points efficiently also in complex scenarios. Explainability of the pathway interactions and how they steer the network is a further strong point of our approach since the influence of the external stimuli can be traced in the network and thus double-checked for plausibility with domain knowledge and targeted lab experiments.

To showcase our pipeline with a modeling process, we employed single cell transcriptome data obtained by Xue JY et al., 2020 from a non-small cell lung cancer cell line, specifically H358, which harbored a Kirsten Rat Sarcoma Viral Proto-Oncogene (KRAS)^G12C^ mutation [62]. Non-small cell lung cancer is a particularly aggressive and often difficult to treat around the world. These data encompass four distinct measurement points taken from the commencement of a therapy with a KRAS inhibitor ARS1620. In a 2018 study, led by [22], ARS1620 exhibited substantial therapeutic potential. However, it's worth noting that in the context of treating non-small cell lung cancers (NSCLCs) with KRAS inhibitors like ARS1620, a significant challenge arose as these tumors frequently developed resistance during the course of treatment [28], [60], [62]. Wu et al., 2022 reviewed all possible proven FDA drug-target therapy options for different tyrosine kinases (ALK, EGFR, MET, RET, VEGFR, and NTRK). Targeting points like ROS1, KRAS, and BRAF are briefly discussed in [60] with regard to the problem of the drug resistance mechanisms in NSCLC. All these findings demonstrate the importance of finding new (combined) drug targets or the most efficient combination of existing drugs targeting specific parts of a complex network of genes.

We remark that our framework considers all modeled network interactions and the joint effect of intervention combinations on them at once which might provide additional insight to considering single intervention points and analyzing their effects separately.

In our study, we have identified the target molecules Aurora Kinase A (AURKA) and Cadherin 1 (CDH1; often named E-cadherin) as a promising candidate for a combined drug target based on the data. Their cooperative action has a synergistic effect on impeding tumor growth, specifically suppressing proliferation, and promoting apoptosis, ultimately leading to cell death according to the analysis based on the data-specific model.

The pipeline DataXflow efficiently not only automates and performs modeling and data analysis tasks, but we offer in addition specific intervention point identification, and explain how the optimal drug target combination can be identified for a given network. Moreover, a framework is provided to apply the (semi)-quantitative SQUAD model to experimental data quantitatively, including an appropriate data normalization. A procedure for an iterative generation of model topologies to overcome unknown regulation relations has been developed that is feasible with highly non-linear model equations, like the SQUAD model. We explain these contributions, among other things, in detail in the following sections. In the Supplement, tutorials with examples how to use DataXflow are provided.

## 2 Methods

In this section, we explain the methods we combine in our software framework and explain how to use them. To contribute to streamlining data-driven modeling, we aim to automate the steps of the process to foster mechanistic modeling and the exploitation of the information encoded in the model to steer the corresponding system in a desired manner.

We describe the data preparation, in particular the normalization required to be compatible with the SQUAD model that we outline as well. We explain the D2D fitting framework in combination with the chi-square test to evaluate the goodness of the fit on a statistical level. Furthermore, we describe the external stimuli framework to determine efficient intervention points in the model that are supposed to steer the model as close as possible to a desired state. There are two different kinds of states. There are states that are temporary and the network only rests in it under the action of the external stimuli [6], like a therapy regulating the consequences of a mutated gene. If the external stimli decay, the current state of the network decays to the ground state. The second kind of a state is called a steady state. This state is a state in which a model rests quite stable even under slight external perturbations [8], like stem cells that have differentiated to different tissue. Once the system has been transferred into such a state due to the action of external stimuli, it rests there, even if the external stimuli decay. The action of the external stimuli transfers the network from one steady state to a different one, called switch. The external stimuli framework calculates the most efficient external stimuli causing this switch.

In the Supplement, the graphical interfaces of JimenaE are explained, how to set up necessary scripts and explain the entry masks as well as the meaning of each parameter that can be set up.

Furthermore, we explain all the helper scripts that we used and how to apply them. These scripts are all contained in the DataXflow pipeline and given in the git repository under https://github.com/MarvelousHopefull/DataXflow.

In Fig. 1, we provide a graphical abstract how the different software tools, explained in the beginning of the Methods section, make the DataXflow.

### 2.1 Data preparation in Python

Single-cell data from H358 cell line, obtained under KRAS inhibition, collected at various time points (0 h, 4 h, 24 h, 72 h), were sourced from Xue JY et al., 2020 [62]. The data, originally presented as log-transformed counts, were subjected to additional pre-processing. In a first filtering step, information about the cell line H358 was distilled out of the extensive dataset since several cancer cell lines were investigated in this dataset [62]. The filter-dataset contains information regarding the H358 cell line, with four files (measurement of time points (0 h, 4 h, 24 h, 72 h) documenting data on gene counts for each individual cell for 8687 genes. For further analysis, the data must be specifically normalized since the SQUAD model, including our extensions regarding external stimuli, expects expression levels normalized between 0 and 1. We explain more in the section about the SQUAD model below.

For addressing the challenge of "dropout" issues resulting in missing measurements, filled in with zeros, it was necessary to eliminate these gaps before proceeding with normalization, meaning deleting these samples from the data set. This approach mitigates the effect of measurement errors modeled by zeros influencing calculations for mean and standard variation. For the normalization procedure, we determined the maximum gene expression value across all cells and time points (comprising 0 h, 4 h, 24 h, and 72 h) for each gene. The final model with the genes of interest consisted of 21 genes and two output nodes (apoptosis and proliferation). Gene counts were only available for 18 genes for this topology.

Subsequently, we divided each gene's expression value (for all cells and time points) by its corresponding maximum value. Through this normalization process, each gene is assigned a value between 0 and 1. A mean value was then determined per gene and measurement time point over all cells, as well as a standard deviation. Thus, each gene has four measurement data points with standard error bars depending on the time. For the implementation of this preparation, two Phyton scripts are provided named by ‘Filtering_script_scH358′, selecting the H358 cell line and time points, and ‘Normalisation_script_scH358′, normalizing the data as described above. Scripts are generated under Spyder 5.3.3 (Python version 3.9) in Anaconda (conda version 23.3.1) and available in the folder “data_preparation” in the git respository.

### 2.2 SQUAD model

We generate the mathematical equations based on SQUAD [31] in an automated manner from an interaction graph. In the Supplement, we show how to use JimenaE to generate them from a graph. An example graph is given in the git repository in the “Example” folder. The SQUAD model equations are used in the calculation within the D2D and external stimuli framework. In this graph, the user sets up the model topology meaning what agent in the model/network interacts with which other agent(s). In our case, we focus on gene regulatory networks, that means we set up which gene activates or inhibits the expression of a gene. However, we remark that our software modules can be extended for equation generation of other kinds of model types that describe the relevant interactions suitably, e.g., Michaels-Menten or law of mass action [9], [7].

**Fig. 2:**
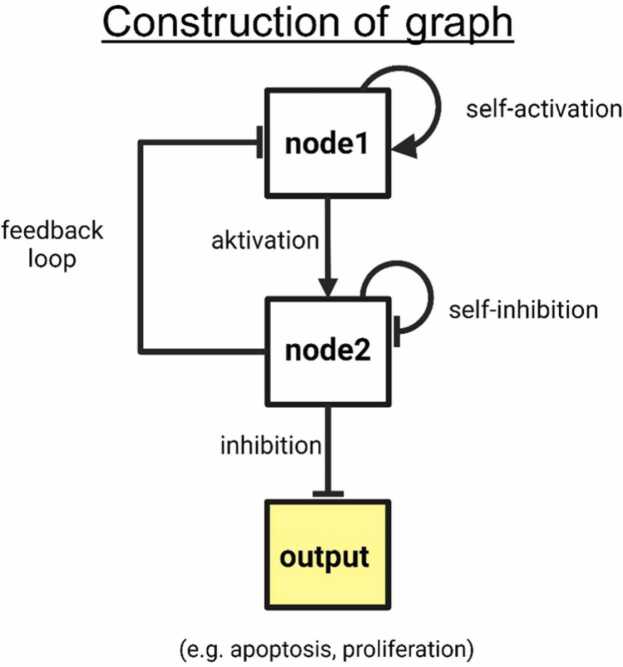
**Construction of graph.** Each node represents a gene, which regulates the activity level of corresponding nodes. The regulation to which a node is subject is represented by arrows, which can cause activation or inhibition. There are 3 components: Activation or inhibition arrows: activation or inhibition of nodes; Self-activation or self-inhibition arrows, meaning a node regulates itself; feedback-loops: The regulation of node influences the node that activated the node beforehand. This influence can be activating or inhibiting. Output nodes reflect the effects of the activities in the regulatory network (e.g. proliferation). Created with BioRender.com.

The graph is set up as follows with the yEd tool. This tool is available under the following link www.yworks.com. Each gene is modelled as a node whose expression (transcription or translation) influences other genes in their expression level, modeled by the activity levels of the nodes (Fig. 2). If the expression of a gene activates the expression of a gene, then a spike-shaped arrow originates in the node representing the gene whose expression activates the corresponding gene and points to the node modeling the activated gene. Analogously, if the expression of one gene inhibits the expression of a gene, then a t-shaped arrow originates in the node which represents the gene whose expression inhibits the corresponding gene and points to the corresponding node modeling the inhibited gene.

From the graph topology, the following system of equations is derived. The system of equations is taken from [31] and is called SQUAD model. The additional term $r_{j}\;$in (E1) is modelled according to the external stimuli framework [6], [8].

(1) $$\frac{dx_{j}}{\mathrm{dt}}=\frac{{-e}^{0.5h_{j}}+e^{-h_{j}\left(w_{j}-0.5\right)}}{\left(1{-e}^{0.5h_{j}}\right)\left(1+e^{-h_{j}\left(w_{j}-0.5\right)}\right)}-\gamma_{j}x_{j}+r_{j}$$

(2) $$r_{j}=\{\begin{array}{l} 0,\text{ if unregulated}\\ \delta_{k}u_{k}\left(1-x_{j}\right),\text{ if upregulated}\\ -\delta_{k}u_{k}x_{j},\text{ if downregulated} \end{array}\;$$

(3) $$\begin{array}{c} w_{j}=\{\begin{array}{l} \left(\frac{1+\underset{n}{\sum }\alpha_{n}}{\underset{n}{\sum }\alpha_{n}}\right)\left(\frac{\underset{n}{\sum }\alpha_{n}x_{n}^{a}}{1+\underset{n}{\sum }\alpha_{n}x_{n}^{a}}\right)\times \left(1-\left(\frac{1+\underset{m}{\sum }\beta_{m}}{\underset{m}{\sum }\beta_{m}}\right)\left(\frac{\underset{m}{\sum }\beta_{m}x_{m}^{i}}{1+\underset{m}{\sum }\beta_{m}x_{m}^{i}}\right)\right),\;\text{if }x_{j}\text{ has activators and inhibitors}\\ \left(\frac{1+\underset{n}{\sum }\alpha_{n}}{\underset{n}{\sum }\alpha_{n}}\right)\left(\frac{\underset{n}{\sum }\alpha_{n}x_{n}^{a}}{1+\underset{n}{\sum }\alpha_{n}x_{n}^{a}}\right),\;\text{if }x_{j}\text{ has only activators}\\ \;\left(1-\left(\frac{1+\underset{m}{\sum }\beta_{m}}{\underset{m}{\sum }\beta_{m}}\right)\left(\frac{\underset{m}{\sum }\beta_{m}x_{m}^{i}}{1+\underset{m}{\sum }\beta_{m}x_{m}^{i}}\right)\right),\;\text{if }x_{j}\text{ has only inhibitors} \end{array}\\ \;\;\;\;\;\;\;\;\;\;0\leq x_{j}\leq 1\\ \;\;\;\;\;\;\;\;\;\;0\leq w_{j}\leq 1\\ \;\;\;\;\;\;\;\;\;\;\left\{x_{n}^{a}\right\}\text{ is the set of activators of }x_{j}\\ \;\;\;\;\;\;\;\;\;\;\left\{x_{m}^{i}\right\}\text{ is the set of inhibitors of }x_{j}\\ \;\;\;\;\;\;\;\;\;\;\sum_{n}\alpha_{n}x_{n}^{a}\text{ summs up all weighted activators of }x_{j}\;\\ \;\;\;\;\;\;\;\;\;\;\sum_{m}\beta_{m}x_{m}^{i}\text{ summs up all weighted inhibitors of }x_{j} \end{array}$$

For a node j with activation level $x_{j}$, the activators and inhibitors of this node are those nodes that have an ingoing activating/inhibiting edge into the node j.

To keep the curves of the activation levels of the nodes between 0 and 1, we set the decay $\gamma_{j}=1$ for all j in equation (E1) and consequently, the decay parameters $\gamma_{j}$ are not part of the optimization variables fitted by D2D. Below, we elaborate more on possible other choices for $\gamma_{j}$ and the corresponding consequences. Furthermore, the superscript ^*a*^ refers to nodes that activate/increase the activation level of the corresponding node j and the superscript ^*i*^ refers to nodes that inhibit/decrease the activation level of the node j. The parameters $\;\alpha_{n}\;$ and $\beta_{m}$ weight the influence of the activation levels of the corresponding nodes on the node j. The parameters $h_{j}\;$ model the non-linearity of the activation behavior of the activation term (first term in (E1)). The activation term is the term that models the effect of other genes on the activity level of the gene j. For details, please see [31]. The sums in the definition of $w_{j}$ integrate all the effects of the regulating genes together. If this number reaches a certain threshold, then the activation term rapidly changes its value for small changes in the activity levels of the regulating genes depending on the values of the parameters *h_j_*. This non-linearity is supposed to model the non-linear regulation behavior of genes. Next, we reason why with our setting the activity levels stay between 0 and 1. If all activity levels start between 0 and 1, then $w_{j}\in [0,1]$. For all activity levels equal 0, $w_{j}=0$ for the only-activator case. Increasing the activity levels results in a monotonic increasement of $w_{j}$ until it equals 1. When increasing $w_{j}$, the nominator of the activation term increases, and the denominator decreases until the activation term reaches its maximum equal 1 since for the denominator holds

$$\left(e^{\frac{1}{2}h_{j}}-1\right)\left(1+e^{-\frac{1}{2}h_{j}}\right)=1-e^{\frac{1}{2}h_{j}}+e^{-\frac{1}{2}h_{j}}-1=-e^{\frac{1}{2}h_{j}}+e^{-\frac{1}{2}h_{j}}$$

for $w_{j}=1$ such that denominator and nominator cancel out, and thus the ratio equals one. If $x_{j}$ equals 1, the decay term equals the activation term such that the right hand-side equals zero (if the external stimuli are not present). However, since in this case the time derivative of the state is zero, the corresponding activity level does not change its value, resting at 1. Maybe the activation term decreases a bit, e.g., due to some activating activity level decreases, then the decay term is bigger, resulting in a negative right hand-side, lowering the value of the corresponding activity level. However, if the activity level is 0, the decay is zero, making the corresponding activity level rest at 0 until the activation term gets positive. Consequently, the activity levels stay between 0 and 1. The other two cases of only-inhibitors and mixed are analog.

The choice of $\gamma_{j}=1$ in this case is rather motivated by computational aspects than biological ones. We see that the developed framework is optimized for normalized activity levels since it unifies the usage of the numerical methods. In case we would not like to normalize the data, it would require it at some points anyway, like in the objective of the optimization problem to rule out the weighting effect of the absolute values of the activity levels. However, we have to make sure, that the model is consistent with the normalized activity level values, meaning the activity levels do not leave their boundaries of 0 and 1. As reasoned above, the activation term can reach any value between 0 and 1 as well the decay term. That means that per see no values for the time derivatives are excluded for our choice of $\gamma_{j}=1$. There is an option to include $\gamma_{j}$ into the optimization process, resulting in different values than 1, while still ensuring that the states stay between 0 and 1. We need to state-constrain the states $x_{j}$ by adding the constraints $0\leq x_{j}\leq 1$ for each j below (E4) [5] to the best fitting procedure. Since D2D solves the corresponding optimization problem for the best parameter fit with MATLAB routines, we could fit the parameters such that the model fits best the data subject to $0\leq x_{j}\leq 1$ for each j by adding these state constraints and choosing a corresponding solution method. Instead of constraining only $\gamma_{j}$ to ensure the state constraints, this method would distribute the consequence of the effects to all parameters. Probably, we would need to extend the external stimuli framework analogously. However, solving state-constraint problem are computational more costly, already by the fact that the optimization algorithm has to consider more constraints, and we will outline in the Discussion that a fast solution method of the best fit is advantageous since we need to solve the corresponding optimization problem several times when adapting the topology iteratively.

The term (E2) models the influence of the external stimuli. The parameter $\delta_{k}$ models the coupling of the external stimulus k and its activity level $u_{k}$ with gene j. For in upregulating external stimulus, the term $\left(1-x_{j}\right)$ ensures that the corresponding activation level is bounded from above by 1 and an active external stimulus has no effect anymore if the corresponding gene is expressed at its maximum. Analogously, for the downregulation where the term $x_{j}$ ensures that the activation level is bounded from below by 0 meaning that the activation level cannot become negative even if the external stimulus is still active. This is reasonable particularly if there is already no expression of the corresponding gene since inhibition should not have any effect any more on that gene. The parameters $\alpha_{n},$ $\beta_{m}$, $h_{j}$ and $\delta_{k}$ are fit by D2D such that the activation levels of the nodes fit the data best.

### 2.3 D2D model fitting

More formally, D2D is a software framework that solves the following optimization problem

(4) $$\begin{array}{c} \underset{p}{\min}\underset{l}{\sum }\sum_{i}\sum_{j}{\left(\frac{x_{l,j}^{i}-{\overset{\sim }{x}}_{l,j}^{i}}{\sigma_{l,j}^{i}}\right)}^{2}\\ \text{s.t.}\;{\overset{˙}{x}}_{l,j}^{i}=f_{j}\left(x_{l}^{i},u_{l}^{i};p\right)\\ \;\;\;\;\;\;\;\;p\in D \end{array}$$

where the superscript i refers to the time point for which we have a best value ${\tilde{x}}_{l,j}^{i}$ of measurements and a corresponding standard error $\sigma_{l,j}^{i}$ for the activity level $x_{l,j}^{i}$ of gene j in experiment l. An experiment to generate the data for the presented framework is defined by a fixed set of conditions, e.g., by the time curves of the external stimuli and the initial values of the states. Each experiment with the fixed conditions is repeated several times such that we can calculate the best values ${\tilde{x}}_{l,j}^{i}$ and standard errors $\sigma_{l,j}^{i}$ from the corresponding measurements from the different instances of the measurements. If, e.g., the concentration of a drug, what the time curve of an external stimuli could model, at one time point is changed compared to a former run of an experiment, then this change defines a different experiment because the administration of the drug is different to the scenario before. This requires that the external stimuli are under a high control and can be reproduced with a very low standard error compared to the standard errors of the measurements of the states. If this is not the case, we should reconsider the model and redesign the model of the “external stimulus” including a potential dynamic that rules the influence of the experimenter and the quantity that influences the network, like a bolus injection (influence of the experimenter) of a drug that is distributed by some dynamic in the system to influence the states. Another example is the administration of a drug with a constant concentration for all time. We repeat this experiment for a fixed concentration several times to generate the data for best values and standard errors and if we change the concentration, then these measurements account for a different experiment. The activity level $x_{l,j}^{i}$ is determined by the corresponding dynamic $f_{j}$, e.g., the SQUAD model, of gene j where the vector $x_{l}^{i}$ contains the activity level of all genes at time i. Analogously for the external stimuli $u_{l}^{i}$. We remark that the dynamic only depends on the gene and not on time or the experiment, meaning the topology is considered as constant in this work. The parameters that can be fit are summarized in the vector p where the values are box constrained by the box D. In our case, the vector p contains all $\alpha_{n},$ $\beta_{m}$, $h_{j}$, $\delta_{k}$ and initial values $x_{l,j}^{0}=x_{l,j}\left(0\right)$ that can be optimized such that the activity levels $x_{l,j}^{i}$ best fit the experimental data $\;{\tilde{x}}_{l,j}^{i}$ evaluated at time point i. We further remark that the parameters in p do not depend on time or the experiment and are considered to be constant in this framework.

In the Supplement, we explain how to use the JimenaE GUIs to set up scripts to implement this optimization procedure into MATLAB scripts that can be executed in the D2D framework and explain their components, in particular how they are related to the optimization problem described above. Furthermore, we explain how to set up the data such that D2D can load it. We particularly explain how to set up the scripts in a multi-experiment setup and additionally the case where the network’s initial states of the activity levels are intended to be varied in different experiments. An example can be the switch from one steady state to another one which could model the transition from (mature) stem cells to specific tissue.

To make the best parameter fit with D2D, we need to execute the following steps within MATLAB. To attain an optimal parameter adjustment within MATLAB, it is advisable to relocate all scripts generated with JimenaE into a newly generated folder within the D2D directory, downloaded from https://github.com/Data2Dynamics/d2d.

**Fig. 3:**
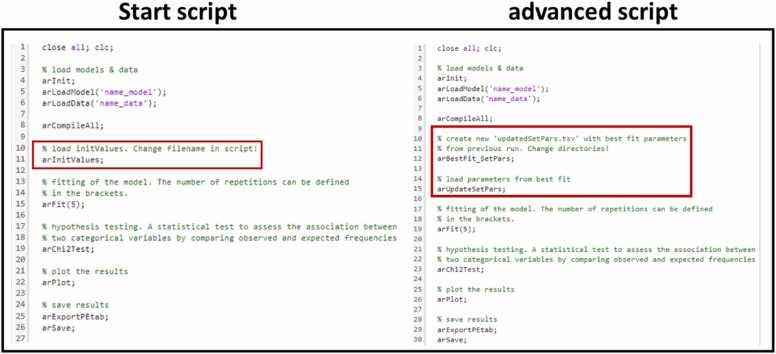
**Setup scripts for D2D.** Start script is required for the first fitting of the model. Advanced script can be run after a first good fitting of the model. The difference in both scripts is the read-in of the initial values. In the start script (left), the starting values are sourced from the outputs provided by JimenaE. In the advanced script (right), we replace these initial values found in the “initValues” file with the best-fit parameters obtained from the previous run, which are stored in the PEtab folder as parameter.tsv. This process generates an updated “update_arSetPars” file. All explanations of the respective functions are included in the text.

For the execution of the fitting process, stick to a specific folder structure. Create a parent folder with dedicated subfolders "Data" and "Models" and insert the specified scripts "start_script" and “arInitValues”. In the "Data" folder, we put the data files containing normalized data (see Methods “Data preparation in Phyton” and Supplement “JimenaE GUI explanation to set up D2D scripts and their description”), typically called "name_data.csv" (an example is given in our git repository in the “Example” folder), along with the corresponding output file “name_data.def” from JimenaE, defining how the data is integrated into the model. For seamless integration, it is crucial that both files share identical “name”. If we run multiple experiments, extend the name with a numerical identifier for each experiment, like “name_data1.csv”, “name_data2.csv” and so on. On the other hand, the “Models” folder only hosts the JimenaE output file named “name_model.def”. To perform the best fitting procedure, the scripts “arBestFit_SetPars”, “arUpdateSetPar” and the advanced setup script “advanced_script” are required in the parent folder instead of the “arInitValues” and start_script. We provide the required scripts in the “D2D_extended” folder in our git repository. Additionally, the other generated files need to be added to the parent folder. To initiate the fitting process, the start_script loads both the model and the data simultaneously, then proceeds to compile (Fig. 3). Further the function “arInitValues” reads the initial guesses of the parameters’ values (sometimes called initial values) to be optimized which are generated in JimenaE automatically. The initial guess values serve as starting points for the optimization routine from where parameters are adapted such that the model output fits best the data. The script “arInitValues” is not included in the original D2D framework and has to be included in the parent folder, as described above. After setting initial guesses for the optimization, the function “arFit” fits model parameters. In this work, lsqnonlin is used on normalized data with standard settings (for more information: https://github.com/Data2Dynamics/d2d and [46], [47]). There are other solvers available from MATLAB used in D2D that one can choose, for details see here. The number of fitting iterations varies for each model. The number of fitting iterations, which are automatically consecutively run, can be specified within the parentheses after “arFit” (Fig. 3). The optimal fit can be identified through the output in MATLAB, specifically by observing changes in the chi-square value, which serves as an objective to be minimized by the parameter adaption of suitable algorithms. The chi-square value, defined by

$$\chi^{2}=\sum_{l}\sum_{i}\sum_{j}{\left(\frac{x_{l,j}^{i}-{\tilde{x}}_{l,j}^{i}}{\sigma_{l,j}^{i}}\right)}^{2},\;$$

is minimized by the parameter fitting procedure of D2D. The smaller the chi-square value is, the closer the model values $x_{l,j}^{i}$ are to the corresponding best value ${\tilde{x}}_{l,j}^{i}$ from the data weighted by the standard deviation, or equivalently, standard error $\sigma_{l,j}^{i}$, also calculated from the corresponding measurement points, please see “Data preparation in Python” for details. The more reliable a best value is (smaller standard deviation), the more the deviation between the model and the corresponding data point accounts for the total chi-square value. Consequently, the parameters will be optimized to minimize the difference between the model and the data points with the highest reliability since the same distance minimization provides a higher gain in terms of a smaller objective (chi-square value) than for unreliable data points. We remark that the index j does not have to run through all the genes but only through those for which there is measurement data.

The level of the improvement of the chi-square value serves as an indicator for the convergence to the best fit of parameters. When the chi-square value experiences minimal change after a run of “arFit”, and thus the improvement approaches zero, it may indicate that the system has achieved an optimal state.

The "arPlot" function generates graphs for each node with corresponding measurements. These graphs display curves and the value of the corresponding contribution of this curve to the total chi-square value, thus helping to rank each node's contribution to the total chi-square value and thus indicating where model improvement is most needed. Depending on this evaluation, adjustments to the model may be necessary. The plot can be saved as an image for example. For a detailed output, one can utilize the "arExportPEtab" function to export the parameter estimation as an SMBL file (https://github.com/Data2Dynamics/d2d/wiki/Support-for-PEtab). The output of “arExportPEtab” is directly located in the parent folder as a folder called PEtab. If the goal is to archive the entire current workspace, the "arSave" function can be employed. Statistical evaluation of the model can be determined with the function ‘arChi2Test'. This function provides statistical results regarding the significance of the observed chi-square value assuming the deviation between data and model are solely from noise. We elaborate more on the rationale of this test in the next subsection.

The key functions essential for D2D can be accessed at this link.

### 2.4 Model evaluation with the chi-square test of goodness-of-fit

In a real scenario, there are measurement errors that come from white noise. Even if our model perfectly fits to the underlying dynamic, the corresponding chi-square value might not be zero due to these errors. We need to evaluate if the errors are caused by noise or rather have a different source like a systematic model error. After fitting the parameters of the model to minimize the chi-square value, the corresponding observed chi-square value might not be zero and we need to assess if the deviation from zero is only caused by noise as follows. Assuming that the difference between model and measurement is just the consequence of white noise, there is the necessity for a statistical test to estimate if the observed chi-square value is unlikely just caused by noise or if we should rather assume another source of the model deviation from the data, like a model misses an important part of the real system. With the chi-square test of goodness of fit, we can estimate how likely it is to get the observed or bigger chi-square value under the hypothesis that the model deviations just come from white noise, i.e., the terms $\frac{x_{l,j}^{i}-{\tilde{x}}_{l,j}^{i}}{\sigma_{l,j}^{i}}$ are each independent Gaussian distributed random variable with zero mean and variance one. In other words, our model is one realization of a random experiment for each i, j and l with corresponding best value and standard error. The probability to get an equal or higher chi-square value is called the p-value. The bigger the p-value is, the more likely it is that the observed chi-square value is only caused by random fluctuations. The distribution of the chi-square values, according to which the p-value is calculated, is obtained as follows. The sum of N∈N squared independent Gaussian distributed random variables is chi-square-distributed with N degrees of freedom. Consequently, the sum of the terms ${\left(\frac{x_{l,j}^{i}-{\tilde{x}}_{l,j}^{i}}{\sigma_{l,j}^{i}}\right)}^{2}$ is chi-square-distributed under our assumption of white noise being the only source of deviations. However, the degrees of freedom of this distribution need to be adapted since not all terms can vary independently as required for the chi-square distribution with N degrees of freedom. The more parameters of our model are fit to the data, the more degrees of freedom are given within the model to adapt to the noise and fit the parameters such that $x_{l,j}^{i}$ equals exactly ${\tilde{x}}_{l,j}^{i}$. To take into account that any model with sufficient parameters might fit the given data perfectly, without describing the underlying dynamics, the number of the degrees of freedom of the chi-square distribution, which provides the p-value of the observed chi-square value, needs to be decreased by the number of parameters that are adapted with the optimization process. These parameters are called free parameters, for details, see also [9]. Decreasing the degrees of freedom of the chi-square distribution relates the observed chi-square value to the scenario of adding less than N normal independent random variables. These less than N random variables are associated with the terms that the model cannot exactly fit just because of setting free parameters accordingly but because the functional properties of the model fit to the dynamics that generate the measured data. If the p-value is above a level of significance, e.g., 5%, meaning that in at least 5% of the cases, the observed or a bigger chi-square value can be achieved by just adding a corresponding number (N minus free parameters) of squared normalized Gaussian random variables, we cannot reject the hypothesis that the deviation between model and data is only noise. In other words, we cannot reject that the model explains the data. We remark that any model that cannot be rejected based on the data can be taken to extract optimal intervention points with the external stimuli framework. The recommended way to falsify models (reject the model based on, e.g., 5%), thus lower the number of models that cannot be rejected and make a more precise modeling, is to build a solid data foundation with many data points with small standard errors. Such a data base sets small boundaries for a model not to be rejected making sure that the remaining models explain the data, i.e., the underlying dynamic, see [9] for a further discussion, in particular Section 4.5 of this reference.

### 2.5 Iterative topology refinement

We remark that in complex cases where the model topology might not be clear from the start, we can begin the modeling with a reasonable subpart of the genes/nodes. We iterate versions until we have found a small core model that fits well to the corresponding data. Once a core model is found, it can be purposefully extended in small steps, e.g., improve the model where the contribution to the total chi-square value is the most. In such a case, we do not have to optimize all the parameters from the scratch but can reuse optimal parameters of parts of the model from former optimization runs that led to good results. We provide scripts to set the parameters of the core model (or the last well working model before the extension) to the optimal values from a previous run such that only the newly added parameters need to be fit from the scratch. The scripts to perform these operations are named "arBestFit_SetPars" and "arUpdateSetPars", given in the git repository under “D2D_extension”. The usage is explained and exemplified in the Supplement “Fitting tutorial”. Using the optimal values, if known, can keep the algorithm close to a good solution avoiding that the fitting algorithm converges to a different local minimum with a higher chi-square value when starting from the scratch or where curves do not fit well. Additionally, to use well working parameters can save computation time by starting already from a good solution.

Alternatively, we can modularize the model, build each submodel and then glue them together via the external stimuli framework. In order to keep complexity controllable, a modularized modeling approach is recommended where we divide the total system into reasonable smaller subsystems that are all improved separately until they meet the accuracy required. The models are then coupled with the external stimuli framework where the output of one model (e.g., the corresponding values of some states which represent expression levels of genes) is the input (via the external stimuli) of the other model. The external stimuli represent, e.g., the gene expression of other genes. The modeling works by given the corresponding gene expression represented by the external stimuli, fit the parameters such that the other genes in the network fit their data as well as possible. The procedure is described in more detail in [9].

Another option can be to start downstream and then build the model upstream through the gene pathways and refine regulation where the most upstream nodes are constant nodes, which means that they have a constant expression value in the lack of an upstream regulation. Building up the model, the nodes with a former constant dynamic are then replaced by the SQUAD dynamic where more upstream nodes with a constant dynamic take the input part then.

To make use of parameters from previous optimization runs or smaller models that are now extended to include further genes into the regulation network, we can employ "arBestFit_SetPars" and "arUpdateSetPars" instead of “arInitValues” (Fig. 3; red box). These two scripts are written in MATLAB, are not part of D2D and have to be integrated into the parent folder in the D2D framework (folder structure described above) to automate the workflow effectively. The “arBestFit_SetPars” function generates a new output_initValues file containing the best parameters from the previous run directly in the parent folder. The data that is used to set the parameter values is from the previous best fit and needs to be stored in the PEtab folder with the file name name_parameters.tsv. Additionally, the corresponding generated file name_initValues.txt is required and should also be in the parent folder. Substituting the default initial values with the best-fit parameters from name_parameters.tsv leads to the creation of a new "update_arSetPars" file. This file is the input for the function "arUpdateSetPars". This function sets the initial values of the parameters to be fit to the best fit values from the name_prameters.tsv file. It is crucial to ensure that the path to include the information about the best fit (name_parameter.txt) and the new initial values (name_initValues) in the function “arBestFit_SetPars” is accurately specified in order to incorporate the file properly.

### 2.6 External Stimuli Framework

Once we have found a well-fitting model applying the D2D framework, the next step is to find out how to influence the model most efficiently to steer the network into a desired state, like a therapy with existing drugs or the identification of potential drug targets. By this procedure, we aim at efficiently exploiting the information based on measured data encoded in the model with respect to the change we would like to inject. The information is encoded in the network topology and the parameters optimally fit to the data. In our general framework, any perturbation to the network is modeled by external stimuli.

The standard optimal control framework for that purpose looks like, analogous to [6], [8]:

$$\begin{array}{c} \underset{x,u}{\min}\overset{T}{\underset{0}{\int }}\underset{j}{\sum }\varrho_{j}{\left(x_{j}\left(t\right)-\theta_{j}\right)}^{2}+\alpha \underset{k}{\sum }u_{k}\left(t\right)\mathrm{dt}\;\\ \text{s.t.}\overset{˙}{x}=f\left(x,u\right),x\left(0\right)=x_{0}\\ \;\;\;\;\;\;u_{k}\geq 0 \end{array}$$

More mathematically expressed, the external stimuli should act such that the states $x_{j}$ are as close to their desired value $\theta_{j}\in \left[0,1\right]$ as possible, respecting the dynamic f and the initial values $x_{0}$ that have been fit to the data. The external stimuli that solve this problem for a sufficiently large α are called optimal or most effective. Notice the vector notation for formulating the dynamic where the vectors x and u contain the following components $x_{j}$ and $u_{k}$ correspondingly. The vector of time derivatives of the states is denoted with $\dot{x}$. The desired values have the purpose to model where the combination of drug(s) (targets) is supposed to steer the network to, for example, to turn a pathological value to a physiological value. In this case, the desired value differs significantly from the activity level of the corresponding node. However, in case there are side-effects that we would like to avoid, we can set the desired value close or equal to the corresponding activity level. To minimize this term, the optimal external stimuli are supposed not to change the corresponding activity level of the side-effect pathways too much. The weight $\varrho_{j}$ models how important it is for the user that the corresponding activity level of this node reaches the target state compared to the others. We remark that $\varrho_{j}\geq 0$ and $\varrho_{j}=0$ if there is no desired target value given for the corresponding node j. For example, if for one solution, the user would like to have one $x_{j}$ closer to the corresponding $\theta_{j}$, the weight $\varrho_{j}$ can be iteratively increased and the optimization can be performed each time to see if the solver provides a different combination of drug (targets) that result in a smaller difference between $x_{j}$ and $\theta_{j}$. The weighting only becomes important if not all desired values can be met with a drug target combination. Solving this optimization problem for increasing α≥0 reveals only the most effective drug targets to reach the goal of driving the states $x_{j}$ close to their desired values where the deviation from the goal is measured with the term $\int_{0}^{T}\sum_{j}\varrho_{j}{\left(x_{j}\left(t\right)-\theta_{j}\right)}^{2}\mathrm{dt}$. The parameter α weights how effective the action of an external stimulus has to be in terms of pushing states close to their desired value to be non-zero where the costs of the action of the external stimuli are measured with the term $\int_{0}^{T}\alpha \sum_{k}u_{k}\left(t\right)\mathrm{dt}$. An external stimulus is only non-zero if its action provides more reduction of the first term than the increase observed for the cost term (second term). Corresponding scripts to solve such optimal control problems are provided with our git repository in the folder “external_stimuli”. The main script generated with our framework just needs to be executed in a folder where all the helper functions from “external_stimuli” are located. We remark that the variables $\theta_{j}$ are not limited to constants. They can be any given function depending on time. The functions can be implemented in the MATLAB function “get_xd” given in the folder “external stimuli” to set the values accordingly. However, for the scope of increasing or decreasing the expression of genes, a constant desired value can be considered sufficient.

Our JimenaE tool generates the corresponding MATLAB scripts including all the case specific information. A tutorial explaining the usage can be found in the Supplement “JimenaE explanation to set up scripts for the external stimuli framework and their description”. Furthermore, we demonstrate in the Supplement “Tutorial for finding most efficient intervention points” how to apply the external stimuli framework to identify most efficient intervention points in a network, like a combination of drugs or drug targets, with regard to an objective, like the deviation of expression values of certain genes.

## 3 Results

In this section, we showcase how to apply the developed pipeline to fit a model to data, demonstrating the workflow, and analyze the resulting model in terms of efficient drug targets. Furthermore, we illustrate how to prove synergistic effects of drug combinations within a model with our developed framework. Specifically, we show that just a higher dosage of one identified drug target cannot achieve the same effect, measured in terms of deviation from the desired state, as the combination of drug targets does.

For this purpose, our developed pipeline requires time resolved gene expression data with best values and standard errors for each best value. Once a model is fit to the data and optimal drug targets are identified, we focus on the explainability of the pathway interactions and network topology. We then study how interventions into the network, like the administration of drugs, steer the network according to our predefined goals. This is a key point of our approach.

**Fig. 4:**
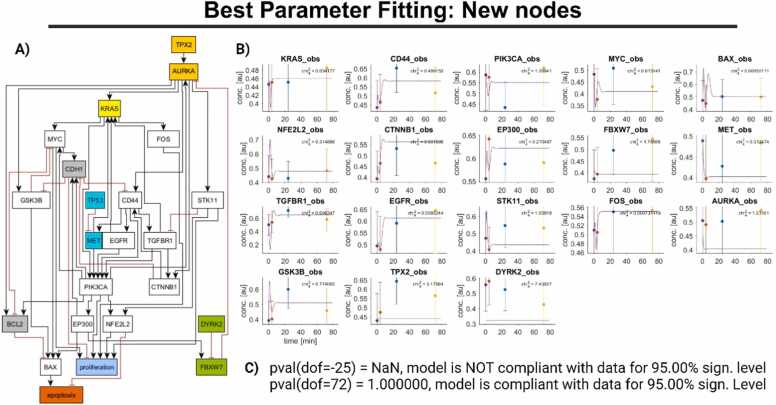
**Final result.** A: The final topology of the protein-protein interaction model after the fitting process. Gray shaded nodes represent missing experimental data, including for TP53 (turquoise). The output nodes, apoptosis (red) and proliferation (blue), are included in this model. B) The adaptation results for 18 genes measured in a protein-protein interaction network are shown. This network consists of 18 regulatory nodes and two output nodes. Each gene is represented by four data points collected at different time intervals: 0 h (purple), 4 h (red), 24 h (blue), and 72 h (yellow). We provided chi-square values for each gene to assess the accuracy of the fit for their expression data. In the protein-protein interaction network, each gene is accompanied by a chi-square value, total chi-square value equals 19.951 with 72 data points and 97 free parameters. C) Statistical test by “Chi2Test” of D2D is shown. The p-value accounting for the measurement points (72) minus the free parameters (97) cannot be defined due to too few data points. Otherwise, any model that would be compliant based on a level of significance (e.g., p-value with data points minus degrees of freedom, abbreviated by dof, greater than 95%) could be taken as an explanation for the data and could not be rejected. Please see the Methods section “Model evaluation with the chi-square test of goodness-of-fit” or [9] for details. Created with BioRender.com.

In the following subsections, we show our final model, including the topology (Fig. 4) with optimal parameters, see the folder in the supplement of this work (PEtab.zip), with respect to our data (data.csv) from [62]. The model with 23 nodes (interacting genes and output nodes) is next analyzed by the external stimuli framework. The MATLAB calculations were performed on the MATLAB software version 2023a. Results are illustrated in (Fig. 6), which is the most efficient anticancer drug combination for a significant reduction in cell proliferation and an enhanced apoptotic effect according to our available data and model. Our calculated drug targets may not be a general combination therapy. Instead, we calculate a drug combination that is supposed to decrease the proliferation and to increase the apoptosis in the modeled cell types. For this goal, we identify the optimal combination considering a user-specified number of drugs and allowed intervention points. Consequently, the calculated drug target combination is supposed to cause these effects in cells of the H358 cell line. In this cell line, CDH1 and AURKA emerged as genes of special significance for therapeutic considerations (Fig. 6). Although the data foundation is sparse in this case, we demonstrate how to apply DataXflow also in such a case, exploiting the sparse information efficiently since the result of downregulating AURKA and upregulating CDH1 might already guide further experiments to try this suggestion. In case of a successful application, e.g., by observing dying tumor cells, the application of our pipeline also to a sparse data foundation can potentially save time instead of trying other combinations and extract the most promising ones with such a data-driven method as presented. We remark that for a rich data foundation the application works identical, however, predictions and results might be more reliable. We provide a description for a data generation that is optimized for the application of our presented pipeline in the Discussion to come to a data foundation that is rich and measurements are set to fulfill the needs of our pipeline.

Analyzing the optimal drug combination in more detail revealed synergistic effects. In particular, testing AURKA revealed a substantial apoptotic effect with no observable response in proliferation (Fig. 7) and CDH1 emerged as anticancer target with a significant decrease in proliferation with no discernible response in an apoptotic effect (Fig. 8). Such non-linear effects, meaning that just a higher dosage of one drug cannot achieve the same effect as two drugs, can be systematically detected by the external stimuli framework, which is very beneficial in networks with many interacting agents where a manual investigation might be very time consuming.

As example for a visual comparison of the different drug therapies, consider activation levels of proliferation and apoptosis when only applying either AURKA inhibition (Fig. 7) or CDH1 (Fig. 8) activation instead of applying both together. Each alone are a suboptimal therapy because they can only fulfill one objective instead of lowering proliferation and increasing apoptosis at once. However, this is the case when administering AURKA inhibition and CDH1 activation together (Fig. 6) as calculated by our framework.

We deliberately choose clear and simple examples to demonstrate our DataXflow framework, but of course far more complex situations (3 or more drugs administered at the same time, 3 or more nodes of interest to treat) can be considered as long as the suitable network is constructed and some data on network nodes are available for a fitting procedure.

### 3.1 Best parameter fit

In the following, we show how we generated a model based on the available data that encodes the information about regulation of the genes and can be exploited with respect to influencing the genes such that the network takes a desired state. The issue is that a complete knowledge about the regulation might not be available or there are contradicting information about gene interactions in the literature making it necessary to iteratively adapt the network topology, and consequently the ordinary differential equations that translate the network topology into a quantifiable system of equation, to obtain a model fitting the data of interest. Our pipeline is optimized for such an iterative modeling procedure since we only need to adapt the network topology, which automatically results in an adaption of the underlying system of equations saving a lot of manual work and making the analysis more resilient against human errors.

In order to be guided where a model needs to be improved most, the framework D2D offers to provide the chi-square value for each observable (gene for which we have measurement data), see, e.g., Fig. 4. With such information, we can purposefully improve the parts of the model that have the biggest deviation from the data. We use the following procedure to iteratively adapt the model.

For fine tuning of the topology, we recommend changing in small steps to check that the solver of D2D does not converge to a totally different solution but only changes in the direction that was intended by the small change. For example, if a gene might need a further activation since its activation level is too small compared with the data, then most of the model’s parameters do not need to be changed. For this purpose, the corresponding initial guesses can be set to a former optimal parameter setting, see the Methods section, in particular the explanations for the scripts "arBestFit_SetPars" and "arUpdateSetPars”. The background is that a highly non-linear dynamic, which the SQUAD model represents, can cause different local optima where a method based on a gradient search can stick and does not proceed to a global optimum. In another scenario, a minor perturbation such as a new activation may induce the convergence to a different local optimum, where curves that were quite fitting after a previous optimization run do not fit any more for the purpose that other nodes fit more. Starting from a former optimal parameter setting can make the convergence behavior more robust allowing us to make incremental improvements to the model topology. The case that the solver converges to a different solution (local optimum) can be identified as follows. There are big changes of many parameters that should not be affected by the small change in the topology leading to time curves of activity levels that do not fit the data that well any more compared to a previous solution before the topology change. In such a case, we should start from the last parameters that have worked associated with the previous topology and only the new parameters should be started from the scratch that are associated with the adaption of the topology. Such a procedure can also save computation time since not all parameters need to be fitted. With the function “arSetPars”, we can set the values of the parameters before fitting in combination with the scripts "arBestFit_SetPars" and "arUpdateSetPars”. Note that of course sometimes starting from the scratch for all parameters after a topology change can lead to a better solution, meaning that the time curves better fit to the data, or the overall chi-square value is smaller since, maybe by the change, conditions are such that the gradient-based method finds a local optimum with a smaller chi-square value. In addition to starting from initial guesses defined by the user, e.g., via our provided GUI, the D2D framework offers a multi-start method where different initial values for the optimization parameters are chosen automatically. To choose this option, we just need to replace arFit by the function arFitLHS. With our pipeline, it is quick to test several options due to automating these steps that are required to start corresponding optimization runs.

The best parameter fit for the model modified from [40], see Fig.sup8 (A), was achieved through a series of iterative steps by D2D using single-cell expression data from [62], depicted in, e.g., Fig.sup8 (B) and given in the data.csv of the supplement to this work, resulting in the topology depicted in Fig. 4 (A). This model focuses on protein-protein interactions and uses gene expression data (72 data points, Fig. 4) to optimize its parameters for a more accurate representation of the biological system under study. In the Fitting tutorial of the Supplement, we provide some intermediate steps from our modeling process to showcase the utilization of our pipeline and give an example how to use the functionalities efficiently to come up with the model depicted in Fig. 4 (A). Specifically, we iteratively changed the topology starting with the one from [40] guided by mitigating the deviations between model and data that contributed most to the total chi-square value.

The fitting process finishes with a chi-square value of 19.951 where the highest contribution to the chi-square value comes from the deviation between the model and the data for TPX2 and DYRK2.

### 3.2 Optimal control and external stimuli to identify effective drug targets

Our best model (23 nodes) is used in a next step in the external stimuli framework to extract encoded information about influencing the network to achieve a high apoptosis and a low proliferation, which is associated with a potential successful therapy. This framework is included in JimenaE to find the best drug targets or combination of existing drugs based on data-driven models. This framework provides an effective way to extract information purposefully regarding the research question. We remark that optimal dosage calculations are included if the underlaying model is fitted accordingly, in particular that the activity levels of the external stimuli represent measured dosages (concentration or amount of a drug).

**Fig. 5:**
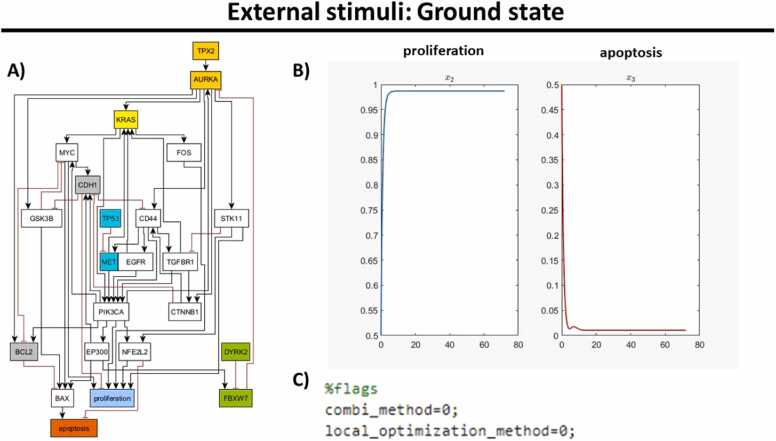
**External stimuli ground state.** A) Best-fitting topology of an NSCLC cell line H358 (Fig. 4). B) The model’s findings reveal an increase in cellular proliferation and a concomitant reduction in apoptotic events. C) Set up flags in the MATLAB script generated by JimenaE to find external stimuli.

Before we run the analysis, the ground state of the output nodes, proliferation and apoptosis, are plotted to compare the changes by the action of the chosen external stimuli (Fig. 5). The curves of the two target states proliferation and apoptosis without any active external stimuli can be obtained by setting the flags “combi_method” and “local_optimization_method” to 0 in the corresponding script generated by JimenaE (Fig. 5(C)) since no optimization is performed and the external stimuli are zero by default. In Fig. 5, we see that proliferation is high and apoptosis is low, which is exactly the opposite of the desired state.

In the present case, we would like to calculate an optimal drug combination or targets, respectively, that provides a low proliferation and a high apoptosis. For this purpose, the mapping file output from JimenaE and the parameter file from the last best fit of the D2D calculations is used to find efficient external stimuli. Apoptosis and proliferation which are included into the model were used as outcomes of therapy success. We remark that expression levels of genes can be targeted directly as well by the corresponding desired values $\theta_{j}$. Apoptosis is a pathway for cell death. A high proliferation represents a dysregulated growth like in cancer. For a successful therapy, the outcome is an upregulated apoptosis and a low proliferation. For our investigation, 14 external stimuli (downregulating: KRAS, MYC, NFE2L2, EP300, MET, TGFBR1, EGFR, STL1K, AURKA, CD44, PIK3CA, FOS and upregulating: CDH1, GSK3B) are set up as possible regions of drug targets, please see Fig.sup4 in the Supplement how to set up the scripts. In general, any node can be selected as a potential drug target, and we can use the presented framework to provide the most efficient combination. However, it should be noted that the selection of nodes may have some restriction. Nodes that directly interact with output nodes (e.g. apoptosis and proliferation) can have a large impact on these output nodes. For this small network, we nevertheless chose nodes of interest that had a direct influence on proliferation, here NFE2L2, PIK3CA, MYC, and FOS. All these genes symbolize pathways that promote proliferation and cancer growth [33], [36], [38], [39], [50]. These genes are additionally connected to other nodes and thus an influence of their expression level has impact elsewhere in the network. To consider all these interactions holistically such that the nodes of interest (proliferation and apoptosis) obtain the desired values is one of the strengths of the presented framework. In general, STK11 is a tumor suppressor gene, but downstream, the NFE2L2 pathway promotes proliferation and suppresses apoptosis [26]. Its true function is not yet fully integrated into the signaling cascade and was not considered here. With our example, which represents only a small network, we wanted to show one way how data-driven modeling works and is a crucial component for automating the construction of such models. Interestingly, these candidates (NFE2L2, PIK3CA, MYC, FOS and STK11) were not identified as best target drugs, although they have a direct impact on proliferation. A reason is the other interactions that might have a bigger impact into the opposite direction regarding the desired values of the nodes of interest. The other target genes EP300, AURKA, CD44 and TGFBR1 also influence tumorigenesis and therefore play an important role in the search for new therapeutic options [15], [20], [35], [40], [49]. FDA therapies are available for KRAS, MET and EGFR, which we mentioned at the beginning [60]. For the two target genes that were upregulated in our example, a study by [25] showed that activation of GSK3b has a positive effect and can inhibit tumor growth. However, this is also due to the interaction of GSK3b with b-catenin and c-Myc. We have considered both cases for CDH1 and explained them in more detail below in the end of the Results section. Some genes are already in focus as therapy options and/or are being tested in preclinical studies or clinical trials, such EP300 and AURKA [15], [44]. In this study to showcase the application of the presented framework, we preselected drug targets according to a literature research identifying genes under study and related to apoptosis and proliferation. However, we remark that we can select other/all the nodes as potential drug targets as well to analyze for most efficient drug targets.

**Fig. 6:**
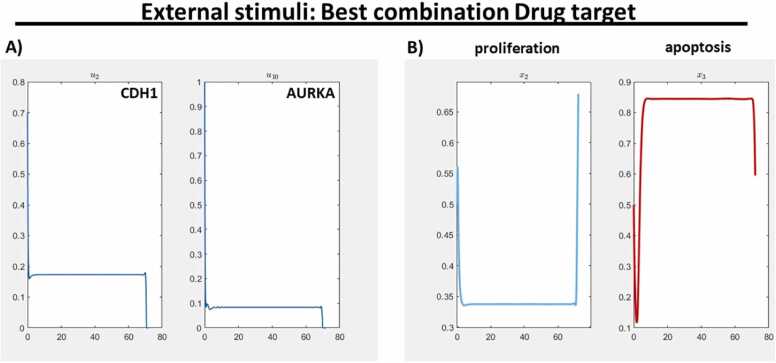
**Result of the optimal control and external stimuli analysis:** The best combination for drug targets according to the available data and model. To get the most efficient external stimuli, an alpha value of 1.0 was set and experiments were conducted on a set of 14 external stimuli. These external stimuli affected genes by down-regulation (KRAS, MYC, NFE2L2, EP300, MET, TGFBR1, EGFR, STK11, AURKA, CD44, PIK3CA, FOS) and up-regulation (CDH1 and GSK3B). A) CDH1 and AURKA were specifically singled out as nodes of particular interest for a therapy and visualized in the graph. All other external stimuli have a constant zero activity level and thus are not plotted. B) Regarding the therapeutic efficacy results, a significant reduction in cell proliferation and enhanced apoptotic effect is observed compared to Fig. 5 (B).

In the Supplement, we provide a detailed showcase how the increasement of alpha leads to a successive decreasing of the number of drugs with a non-zero activity level. For alpha equal 1.0, only two from the 14 external stimuli are left turning out to be most efficient to steer the network in our favor. The result is a combination of CDH1 and AURKA. The influence of these genes is one of the best candidates of a combination treatment option which has a high effect on upregulation of apoptosis (red) and downregulation of proliferation (light blue) (Fig. 6(B)). In the Discussion, we validate these findings with a literature research where AURKA and CDH1 are reported as promising drug targets. However, to the best of our knowledge, the suggested combination has not been investigated yet.

**Fig. 7:**
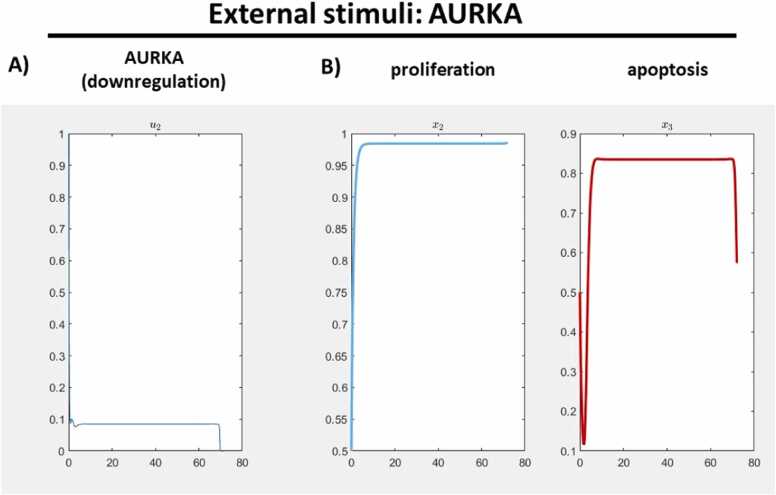
**Result of one external stimulus, only on AURKA.** For an alpha value of 1.0, experiments were carried out with KRAS and AURKA both set to a down-regulated state. A) AURKA emerged as a node of specific interest for therapeutic considerations and is graphically represented. B) The therapeutic outcomes revealed a substantial apoptotic effect with no observable response in proliferation.

**Fig. 8:**
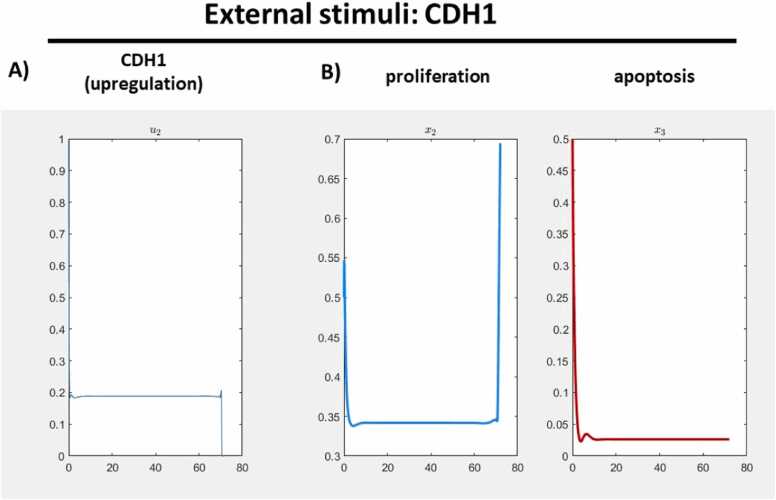
**Result of one external stimulus, only on CDH1.** To explore the impact of external stimuli, we standardized the alpha value to 1.0 and conducted experiments with a set of two external stimuli: KRAS (down-regulated) and CDH1 (up-regulated). A) CDH1 emerged as a noteworthy node for therapeutic considerations and is visually represented in the plot. B) The therapeutic outcomes revealed a significant increase in proliferation with no discernible response in apoptotic effect.

Next, we would like to analyze the effect of the application of either the downregulating AURKA or upregulating CDH1. From the difference of proliferation and apoptosis to the application of both drugs at once, we can conclude synergistic effects. In order to have a comparable weight of an activity of an external stimulus, the alpha value is set to 1.0 for these investigations, which cancels out variations on the time curves or the activity of the external stimuli caused by a variation of alpha, and thus the changes of the time curves of proliferation and apoptosis are from the external stimulus itself when comparing the time curves between different scenarios. The outcomes are depicted in Fig. 7, Fig. 8. To test the single effect of either inhibition of AURKA or activation of CDH1, we have only chosen inhibition of KRAS (which was the reference administration for generating the data) and the other external stimulus each. As expected, the inhibition of KRAS is set to constant zero since for alpha= 1 its action is not sufficiently effective in terms of providing desired changes in the activity levels of the nodes of interest compared to the other two drugs each. For comparison, keeping the alpha value constant is important since the value effects the number and time curves of the non-zero external stimuli. Furthermore, we remark that we do not need to include the KRAS inhibition, and it can be excluded when setting up the corresponding script in JimenaE.

The findings indicate that inhibiting AURKA leads to an upregulation of apoptosis (Fig. 7 (B)), yet there is no discernible change in proliferation when compared to the baseline (Fig. 5). Since the time curve of the activity level is optimized to achieve the best possible objective, meaning a high apoptosis and a low proliferation, we cannot find another administration of the single drug (in particular not a higher dosage) that might have also a lower proliferation (unless we might not have converged to an optimum with the lowest objective value).

The outcome involving CDH1 instead of AURKA presents a contrary scenario, proliferation experiences a downregulation, with no observable response in terms of apoptosis, compared to the result for AURKA for the nodes of interest, see Fig. 5 and Fig. 8 (B).

These last two results from the respective single influence on AURKA and CDH1 show that a sole change in the regulation of only one of these drug-target genes does not achieve a complete therapeutic success, apoptotic response, and inhibition of proliferation. A combination therapy with AURKA and CDH1 is thus recommended for a promising prospect of a successful therapy from our numerical investigation based on the used data. Even in a sparse data foundation, as in the presented work, it might already be helpful to extract the information available to design new purposeful experiments efficiently.

Given that alpha remains constant at 1 for all experiments, we have ruled the effect of the alpha values out influencing the time curve or activity of the external stimuli by weighting their activity against their effect on steering the nods of interest close to their desired values.

As the last experiment, we evaluated if CDH1 is better up- or downregulated by putting both an activating and inhibiting external stimulus on CDH1. Since there are different suggestions to different cancer types in the literature [14], [37], [56], [64], we would like to showcase how to use our pipeline to find data-driven a decision for the given data and thus cancer type. We have the following set of external stimuli downregulating KRAS, MYC, NFE2L2, EP300, MET, TGFBR1, EGFR, STK11, AURKA, CD44, PIK3CA, FOS, CDH1 and upregulating CDH1, GSK3B. For alpha= 1, we repeat the calculation of optimal external stimuli. Based on our data and model, we clearly have the best effect if CDH1 is up regulated since the downregulating external stimulus is set to zero, meaning the downregulation has not an efficient effect regarding our aim of a low proliferation and a high apoptosis in this case.

## 4 Discussion

In the presented work, we demonstrated how to synergize data-driven modeling, best parameter fit and optimal control to extract information from experimental data that guides further experiments for a specific research question. Applied to clinic, fitting models with patient-specific data, also including side-effects, may even pave the way to a new level of optimized therapy and personalized medicine. A vision of a systemic chemotherapy is mentioned in [30] including bringing toolkits to the clinics to design patient-specific systemic therapies. However, to automate well understood modeling processes and to generalize accordingly to a stable solution requires further steps to which we would like to contribute with this work.

Our results show how to obtain the best parameter fit applying D2D, using our framework for iteratively constructing a model, if necessary, postulating the interacting of genes guiding an efficient research for unconsidered important genes for the concrete modeling. The synergy of our selected methods is demonstrated, analyzing the best fitting model (23 nodes) by the external stimuli approach in our DataXflow framework to find best intervention points. This means to reveal best anticancer drug combinations (Fig. 6), which targets AURKA (Fig. 7) and CDH1 (Fig. 8) to influence proliferation and apoptosis in the opposite directions compared to the untreated scenario. Our methods are able to operate with both limited and rich data. This is one of the strong advantages of our approach extracting the information from the data as well as possible given the data foundation to guide further research directions efficiently and based on data.

Our framework is able to consider side- and therapeutic effects by setting desired values for specific nodes, e.g., to consider side effects that a gene expression should not be changed, the desired activity value can be set to the value the gene has in an untreated scenario. Since the outcome of the model generation is a network, the analysis results for drug targets are explainable, and not just the answer of a black box, by considering the pathway interactions and how the identified interventions steer the network. To build our pipeline, we integrated the software tools D2D [46], [47] and the external stimuli framework [6], [9], [8] into JimenaE [24], which is the hub of our total pipeline, called DataXflow.

Building a data-driven model is an effective approach to extract and explain information hidden in measurement data. The aim of the work is to simplify the process of designing such a model by integrating different tools and automating tasks that come combing the mathematical tools. The advantage is that data-driven modeling from own data can be performed by a bigger audience since the need for specific knowledge in bioinformatics or mathematical expertise is reduced. This facilitates a systems biology perspective in the form of models and thus makes the insights accessible to a broader audience. Our approach showcases how optimal control can leverage the knowledge from a model by extracting targeted information for inducing a desired change most efficiently.

### 4.1 Data generation optimized to apply DataXflow

Specific data is a prerequisite as well as essential for the modeling process and an important part of our approach to join data generation with its analysis. The data acquisition is a fundamental step of the process. It might be that a large number of parameters needs to be determined. For this purpose, it is beneficial if the number of data points scales accordingly, since the larger this amount of data points is, assuming not an increasing standard error, to determine these free parameters, the more precise models can be tested increasing their probability that their predictions work. In order to get enough data, we can measure the data in the following way to obtain a data set that can be used to fit the model efficiently.

We fix a set of external stimuli that we apply during the experiment and in what manner (e.g., intensity and time curve) they are applied. We remark that here the term external stimuli refers to the real stimuli applied in a lab experiment and not to the mathematical representation. Then, we can measure the states, e.g., gene expression, at several points in time between the start and the end of the experiment. Since there is variance in the biological and the measuring process, we need to repeat this experiment more often to obtain a best value with the standard error for each point in time. Measuring for more time points scales the number of data points but not the number of free parameters of the model and thus measuring the states is efficient regarding to our analysis framework. Apart from measuring more data points in time, we can perform measurements for several combinations of external stimuli, defining further experiments, meaning applying different combinations, intensity, and time curves of the external stimuli, if possible, in the lab to generate more data. Following this procedure, the number of free parameters stays the same when the data is included into the model, since the entities that are in the model do not change, but the number of data points (best values with their standard error) increases with each point in time or with different combination of external stimuli, like drug targets, where a measurement is performed. Even the variation of different strengths (e.g., drug concentration or intensity of a physical stimulus) of the external stimuli is another combination that contributes to the number of data points but not to the number of free parameters. Even if measurement is not possible except at the beginning and the end of the experiment, different combinations (different strength or different time curves) of external stimuli can scale the number of data points.

Combining data from different sources (experimental settings) might be tricky, since relevant conditions, which are significant for the outcome of the experiment, like cell lines, used kits or even temperature under which the experiment was performed, etc., might be different. This variation in the conditions, which might not be covered in the model, e.g., constant reaction parameters that should change with the temperature, could lead to an increased variance and thus hypotheses can be less precisely tested.

### 4.2 Modeling heterogeneous tumors

The SQUAD model averages the interaction network over a class of cells. In order to cope also with a heterogeneous tumor, single cells could be clustered according to similarity in their expression profile, see, e.g., [29] and a model can be built for each cluster taking only the data (single cells) into account for each model associated with the corresponding cluster. We start with a model for one cluster by fitting the corresponding data. If the associated topology does not fit the data associated with another cluster just by adapting the parameters, then the changes of the topology that need to be made such that the corresponding model fits the data associated with the other cluster may provide a hint what is different in the other tumor cluster and what might account for the heterogeneity. We remark that also in case of no topology changes are needed, changes in the parameters between the models of the different clusters might give a hint for the heterogeneity. However, also the other way around is possible by analyzing differences in the expression profile of single cells between different clusters, e.g., as in [11], [45]. These works describe pipelines extracting the most relevant differences in the expression profile via a small set of genes characterizing the differences between single cell clusters based on feature reduction and statistical methods. This information can then provide hints what changes in the topology might generate a suitable best parameter fit based on a new topology on a different set of data associated with the corresponding other cluster. By considering the differences between clusters, it could be avoided to start modeling from the scratch for each cluster benefiting from previous knowledge.

In the regime of tumor modeling, there are other directions focusing on the modeling of the tumor’s evolvement [2], [48], [58]. We extend these perspectives by providing a tool for exploring the regulatory network within tumor cells to explain the expression data directly.

### 4.3 Discussing the generation of the model from a biological perspective

In our example, we try to find the best model based on the H358 cell line, non-small cell lung cancer cell line. Regrettably, the data situation was sparse, even though we were able to utilize measurements at four distinct time points per cell (0 h, 4 h, 24 h, and 72 h) and taking only representative genes for the modeling into account generating an effective model. However, the data was recorded only for one drug administration. Data were obtained using single-cell sequencing and were provided by the authors as log-transformed counts [62]. This count table was highlighted by a high amount of nulls also called “droupout-events” [1]. To overcome this problem all nulls were excluded for normalization. We remark that this issue is frequently a topic of discussion and represents a notable drawback of single-cell sequencing. Xu and collogues pointed out further approaches in their study to address such kind of data gaps in single cell RNA sequencing [61]. However, it's important to note that expanding the topology with actual connections remains a time-intensive task, primarily reliant on literature research to this point in time. Such a research was performed during the modeling process where we identified three genes whose action made a better model fit: Microtubule Nucleation Factor (TPX2), Tumor protein 53 (TP53), and Dual Specificity Tyrosine Phosphorylation Regulated Kinase 2 (DYRK). According to the existing literature, it is established that TPX2 activates Aura Kinase A (AURKA) [15], while DYRK2 exerts an inhibitory influence on F-Box And WD Repeat Domain Containing 7 (FBXW7) [23]. The available data aligns with these expected effects in the model fitting. Moreover, [65] emphasized the regulatory role of TP53 in Hepatocyte Growth Factor Receptor (MET) expression. Consequently, despite the absence of available data for TP53, we successfully incorporated these genes into the model, as illustrated in the Fig. 4.

### 4.4 Discussing the resulting drug target combination from a biological perspective

Our research into identifying new target genes for lung cancer therapy within the framework of external stimuli has brought to light two prominent candidates: AURKA downregulation and Cadherin 1 (CDH1) upregulation, also known as E-cadherin (Fig. 6). These genes have emerged as noteworthy options for targeting. According to our calculations, the simultaneous modulation of these genes exhibits a synergistic effect, effectively reducing proliferation and promoting the activation of the apoptotic pathway (as depicted in Fig. 7, Fig. 8). These findings contribute to a deeper understanding of the interplay between AURKA and CDH1 in influencing cellular processes. In the literature, such a synergistic effect of CDH1 and AURKA has not yet been described as combination therapy to the best of our knowledge. Only the individual view is described. AURKA was shown to be the most promising combination with ARS1620 (KRAS-Inhibitor) therapy option in a previous study that was presented by Peindl et al. [40] and AURKA is often discussed in the literature as promising drug-target point in cancer therapy [15], [52], [63]. The role of downregulation for CDH1 has been frequently described in the literature as an effective method for tumor control [64]. In our case, we conducted an analysis on drug targets, both upregulating and downregulating, for CDH1. Our framework identified that the most effective approach for minimizing proliferation and maximizing apoptosis is the upregulation of CDH1 based on our available data. In Liu, Kang, and Tang [28], compelling evidence has been presented regarding the relationship between CDH1 (E-cadherin) and miR-25 in the context of cancer. Their study highlights that when miR-25 levels are elevated, as observed in H358, it results in the downregulation of CDH1. This downregulation of CDH1, in turn, is associated with increased cancer cell migration and invasion. These findings can hold significant implications for achieving therapeutic success for upregulated CDH1 and supports our thesis that an upregulated CDH1 is advantageous for a therapy.

### 4.5 Alternating lab experiments and data analysis is beneficial

In a biological and clinical context, we provide a new method to extract promising cancer treatment options from available data using best parameter fitting and optimal control combined with a user-friendly software automation of certain tasks. This approach serves as a pioneering pathfinder towards discovering data-driven solutions for novel therapeutic targets. Successfully validated targets accelerate the drug target identification by guiding further experiments for a targeted improvement of models and just verifying the suggested drug targets in an experiment instead of trying out many options in lab experiments and canceling candidates experimentally out. We remark that after an experiment, results can be included by fitting the model and extending it to the now bigger database. With such a framework, we are able to iteratively improve a model and guide experiments dynamically, making sure to make always best-informed decisions so that only few experiments are needed.

### 4.6 Further research for supporting and automating topology generation

To facilitate the literature search to generate a potential topology for a scenario, further extensions may include core methods of artificial intelligence, such as natural language processing, to screen publications for reported interactions of genes to accelerate getting ideas for a well-fitting model topology. One could take a chatbot or conversational AI, like ChatGPT, which is based on a large language model and possibly specifically trained or finetuned on (up-to-date) texts about life science. This conversational AI could be asked “I have cells of tissue [tissue name], tell me all genes that are (directly) regulated by gene [gene name]” or “I have cells of tissue [tissue name], tell me all genes that regulate gene [gene name]”. The corresponding suggestions can be tested with our framework by testing which is fitting best given the data we have.

To enhance traceability of the suggestions for the relevant genes, publications can be cut into parts, embedded with a text embedding method, like the text-embedding-ada-002 offered in Python, and the text with its embedding can be stored in a (vector) database. Depending on the context of a question, related text parts can be identified via the corresponding embedding and input into the prompt together with the question as background from which a conversational AI generates its answers. The technique is referred to retrieval augmented generation (RAG) and facilitates the traceability of a chatbot’s answers. By updating the database, the model can provide the up-to-date sources on which it bases its decisions and explanations to check for plausibility and context.

Alternatively, to sourcing gene interactions from text, corresponding gene interaction databases can be used, like STRING or QIAGEN to find connections between genes. Additionally, a pure database search can be combined with the previous approach by feeding this information as well into the prompt of the conversational AI.

Given the genes that should appear in the model, e.g., since they fit to the context for which a model is needed, the interaction graph can be automatically figured out from the references how genes interact. However, sometimes information of regulation might be contrary from different sources and thus there are options for the topology. Our framework of automatically generating equations and fitting a model to the data can be used to select the model best fitting based on the available data, evaluated, e.g., on the chi-square value with decisions for a fitting model based on the corresponding p-value. However, the ambiguity should not appear too often because the number of possible combinations might become too many for trying all combination due to combinatorial and computational time issues.

We remark that with conversational AI pipelines like, e.g., Langchain, there are dedicated tools for using large language models and conversational AI combining them with other tools that are specialized for a task, like model fitting. If a chatbot detects ambiguity, it can call a tool like DataXflow that can fit a model to given data and based on the return value, like the chi-square value and the p-value of each topology version, the chatbot can take further actions to build an appropriate model topology that fits the given data. Furthermore, since D2D returns a chi-square value for each gene, ranking parts of the model that do not fit so far, the chatbot can automatically search for improvements for those parts. The chi-square value would guide the model how to proceed efficiently in case a model topology does not fit and automate the procedure we show in our fitting tutorial in the Supplement. As a vision, such a framework could even automatically answer questions like “Given this data set, which are good drug targets to achieve a successful therapy?”. Also, for this task, the chatbot can call our pipeline step by step for the dedicated analytical tasks, e.g., D2D for model fitting and the external stimuli framework to analyze for the corresponding drug targets once a fitting model has been generated.

In terms of explainability, this described framework is very interesting since artificial intelligence is used to construct a network topology as automated as possible. The network topology is understandable to humans to get insights into the data set and the relations of genes. In addition, drug target or combination recommendations from such a pipeline, e.g., for a therapy can be understood by humans via this network topology which is proved by fitting a model to data. The contrast is where a machine learning model is constructed that takes decisions for, e.g., given the patient-specific data what the therapy recommendation is. Since such a model might be a black box, this approach might lack transparency compared to the approach described above resulting in an understandable network topology. Such an approach can also be a safeguard against hallucinations that chatbots sometimes suffer from.

### 4.7 Further model types to generate automatically

Further extensions will implement more diverse model types apart from the SQUAD model, since the choice of the basic equation type can also influence how good a model fits the data. Further model types foster the application of our framework outside of gene regulatory networks where maybe equations from the law of mass actions or/and Michaelis-Menten-kinetics [9], [7] are more suitable or a model is a combination of several model types, all automatically generated from a model topology. For complex systems from ecology, even other equation types may be necessary to model the relations in such a system effectively.

Furthermore, partial differential equations are a used tool to model tumor evolution [34], [3], [41], [4], [55].

Our git repository allows an easy extension of the software suite for further developments and automations settin up the model topology. The repository is available under https://github.com/MarvelousHopefull/DataXflow.

Since our software tool is modularized, there is the option to test if the rigorous optimal control approach extracts useful information from models fit in other studies or other model types with a low effort before one can develop the automated generated of the corresponding kind of equation types. We can exchange the model equations developed in, e.g., [32], [57] in the script exported from JimenaE for the drug target identification. If we define appropriate external stimuli in that models that an experimenter can influence, then we can test how the optimal control framework fits to the corresponding model type and if the optimal external stimuli are helpful to design promising follow up experiments or give interesting insights.

Furthermore, independent of automating the model generation, it might be beneficial to also provide an interface of the optimal control framework to include already developed models with a low barrier to extract information from a model how to steer it with respect to the desired outcomes. For example, if a model describes the transition into different tumor states [43], the question is how to influence this system optimally such that most tumor cells transit into a beneficial state which can be a concept for a therapy.

### 4.8 Modeling side-effects

A further research direction is to extend the pipeline’s capability such that side-effects can be investigated even better. For this purpose, in addition to the model of pathological cells, a model for the healthy cells is generated. Then, the external stimuli effect not only a single node in the pathological model but interacts with the corresponding node in the model for the healthy cells as well where a drug has also an unavoidable effect in the healthy cell. Both models can be separately fitted to data, however, the current pipeline does not allow to load both models for the optimal control framework and link one external stimulus to several nodes. Analogously, a single external stimulus cannot be linked to more than one node at the moment via the GUI although from the mathematical concept itself there is no limitation and all could be done manually reported so far in this paragraph. Having both models, one for the healthy and one for the pathological cells, the objective might be to change some activity levels in the pathological cell, like the proliferation from high to low and apoptosis from low to high, while keeping important activity levels in the healthy cells almost unchanged to keep side effects low, e.g., a low apoptosis in healthy cells. Keeping expression values in the healthy cell model almost unchanged is achieved by setting the desired gene expression values ($\theta_{j}$) to the physiological ones, which are the values in the healthy model, since deviations from these values lead to an increasing objective. As the topology due to, e.g., mutations, should be different between healthy and pathological cells or in case not, corresponding fitted parameters are different, our framework offers an effective way to find the right combination of drugs fulfilling the objectives as much as possible also in complex network scenarios automatically and exploits the differences beneficially.

### 4.9 Improvement of current implementation

According to our experience, a fast-fitting tool is important because in the generation of the model the fitting routine will be used several times, especially in the case of an unknown regulation where the topology is iteratively refined to come to the final model best fitting to the data. One approach is to optimize the framework that after changing something in the model topology only the affected parts are recalculated. This is a further direction for future research to be integrated into D2D or into JimenaE.

## 5 Conclusion

This work focused on providing a software tool for data-driven modeling and extracting information based on data for steering a system in a desired manner, like a therapy. For this purpose, several software tools were integrated into one pipeline automating tasks that come with combing different tools. An intuitively designed graphical user interface, fine-tuned for the intricacies of data-driven modeling, facilitates an entry into this domain, even for individuals without programming expertise. The intention is to also enable an audience outside the bioinformatic community to model data-driven and to extract information purposefully from the measurement data.

The DataXflow approach contributes to a patient-specific cancer research by synergizing data-driven modeling with the attainment of the best parameter fit and optimal control. The pipeline, as exemplified in constructing a lung cancer model from single cell data, showcases its efficacy in identifying promising drug targets.

We provide a detailed explanation of the mathematical concepts, a software pipeline via a git repository, containing an executable file, together with tutorials how to use the software GUIs and how to apply the methods to the fitting task and network analysis task to search for optimal intervention points, all exemplified with our lung cancer model. Furthermore, we showed how to analyze synergistic effects of a drug (target) combination. Applied to clinic, this will pave the way to a new level of optimized therapy, personalized and precision medicine.

## Funding

Deutsche Forschungsgemeinschaft (10.13039/100004807DFG), grant 492620490 – SFB 1583 /INF (TD, TB; for decision process analysis), Land Bavaria (contribution to DFG grant 324392634 - TRR 221/INF) (TD, TB: for cancer cascade analysis). 10.13039/501100007440Hans-Böckler-Stiftung (grant 415523 awarded to SC).

## CRediT authorship contribution statement

**Tim Breitenbach:** Writing – review & editing, Writing – original draft, Supervision, Methodology, Formal analysis, Conceptualization. **Samantha A. W. Crouch:** Writing – review & editing, Writing – original draft, Investigation, Formal analysis, Data curation. **Jan Krause:** Writing – original draft, Software, Methodology. **Thomas Dandekar:** Writing – review & editing, Writing – original draft, Supervision, Funding acquisition.

## Declaration of Competing Interest

The authors declare that they have no conflict of interest.
