# Supplementary material for: DataXflow: Synergizing data-driven modeling with best parameter fit and optimal control – An efficient data analysis for cancer research
Source: Comput Struct Biotechnol J. 2024 Apr 8;23:1755–72. doi: 10.1016/j.csbj.2024.04.010 (PMC11068525; doi:10.1016/j.csbj.2024.04.010)
Supplement: Supplementary file 1 — Supplementary material. [file mmc1.pdf]

Supplement

We provide further information about how to use the graphical user interfaces (GUIs) of JimenaE, how to use our software for fitting models adapting topologies and use the optimal control framework for identifying efficient drug targets.

JimenaE GUI explanation to set up D2D scripts and their description

Once a graph, representing the regulation topology, is set up, we use this information to generate the SQUAD equations that model a dynamic gene regulatory network automatically with the JimenaE framework. In order to generate the D2D script to determine parameters of the SQUAD model fitted according to the data, we use the following JimenaE interface:
First, we have to load a network file (graph) into JimenaE. In the menu “Network”, we can select “Import yEd File” to choose a “.graphml” file that represents the network topology (regulation information).
Afterwards, we open the GUI by selecting “D2D” in the “Analysis” menu.
The GUI is next explained step by step.

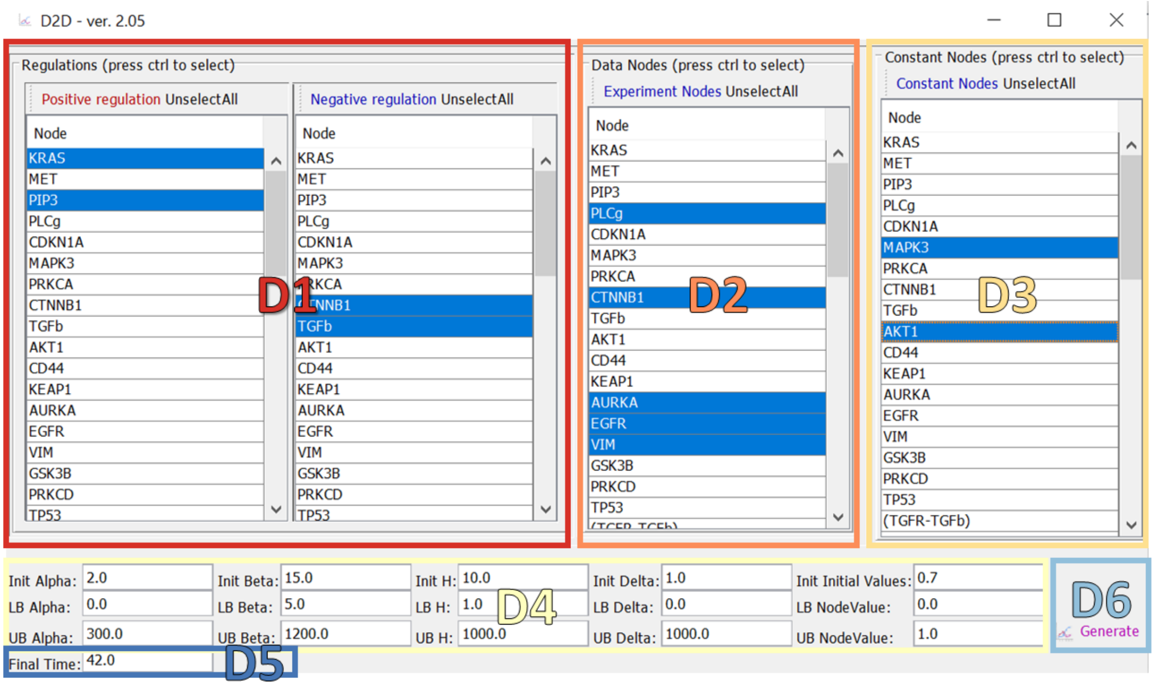

Figure sup1: GUI for creating the D2D relevant files, see text for an explanation of the purpose of those files (GUI1)

The fields D1 to D3 allow us to select specific nodes for different purposes.
D1: Nodes that are up or down regulated by an external stimulus. By “positive regulation”, we add the term $+\delta_k u_k(1 - x_j)$ to the corresponding j-th equation (E2). The mapping between gene names and the index j is provided in the D2D script that is output by pressing “Generate”. Analogously, when marking a gene in the “Negative regulation” column, the term $-\delta_k u_k x_j$ is added to the corresponding j-th equation (E2).
We remark that any list of nodes, equations or any numerical representation of nodes follows the same order as in the original network file. This might differ from the representation order used in other parts of JimenaE, as sometimes an alphabetic listening of nodes is used.

D2: By marking gene names in the column “Experimental Nodes”, we select the genes for which there is measurement data available. This data is used in the D2D fitting process to optimize the parameters of the model. The selection is necessary to generate the D2D scripts accordingly. We remark that not for all nodes in the network data is required but the more nodes have data, the more precise hypotheses can be tested.
D3: Nodes that should have a constant value. The corresponding right hand-side in (E1) is replaced by 0 such that only the initial value can be varied. Such a constant node dynamic is useful if a node is an entry node of the network and has only outgoing edges since otherwise the decay in (E1) would lead to a zero-activation level which could deactivate subsequent nodes as well.
D4: Here we can specify different parameters of the equation. Giving them upper and lower bounds and initial values will help to narrow down the search for best fitting parameters done by D2D. The initial guesses for the parameters, which are then gradually optimized by the optimization procedures, are given in the fields denoted with “Init …”.
D4.1: Alpha: The weights for the activators, denoted with α_n in the equation (E3). In the D2D script, the notation is as follows alpha_i_j meaning that node i activates node j.
D4.2: Beta: The weights for the inhibitors, denoted with β_n in the equation (E3). In the D2D script, the notation is as follows beta_i_j meaning that node i deactivates node j.
D4.3: H: The gain of the differential equation, denoted with h_j in the equations. It means how non-linear the activation potential is, see SQUAD details in (Mendoza and Xenarios 2006) for an activation level for different values of h_j. In the scripts, the variable is named by h_j.
D4.4: Delta: The coupling coefficient for the terms modeling the external stimuli influencing the network. In the equation (E2), these coefficients are denoted with δ_k. In the scripts, the variable is named as delta_k. The k stands for the k-th external stimulus of the network.
D4.5: Init Initial Values: It is the initial value of the variables x_j, which is necessary to provide since the dynamic is modelled by ordinary differential equations. Since the data is normalized such that expression profiles range between 0 and 1 (0: no expression, 1: maximum expression present in the data), upper and lower bounds of the initial values should be kept within [0,1]. In the scripts, these variables are denoted with init_xj.
D5: Enter here the duration of the experiment that your experimental data is based on. The time unit is arbitrary but then holds for the units of the fitted variables that include time, like a rate.
D6: This button will allow us to create the files as specified through points D1-D5. It will open a window, allowing us to choose a name and location for the newly created files.
Several files will be created. Each file description has an exemplary name.
Mapping file: “name_D2DMapping.tsv”: Mapping the parameters to the nodes. It is not needed for D2D application but needed later for another GUI in JimenaE that generates the MATLAB script to determine optimal external stimuli to steer the model into a desired state or behavior, respectively. We will go into more detail below.
D2D model file: “name_model.def”: Needed for the D2D application, holding the information about which nodes exist in the graph, the regulated nodes, the equations for each node and within the equations the role of the different parameters. Apart from the regulated nodes, this information is automatically taken from the graph provided to JimenaE.
D2D data file: “name_data.def”: Needed for the D2D application, holding the information about the time curves of the external stimuli (D1) and for which nodes there is experimental data and measurement errors provided (D2).
D2D Initial Values: “name_initValues.txt”: Needed for the application of D2D, holding the information about initial guesses and upper and lower bounds for the different parameters for

the optimization procedure to fit the parameters best to the data, as selected in the initial values section (D4). For each parameter, an arSetPars('label', init_Value, qFit, qLog, lb, ub) function is listed. With 'label' holding the parameter name, init_Value the initial assignment, qFit is fixed, fitted or constant, here always fitted, qLog is normal or logarithmic, here always normal, lb the lower bound and ub the upper bound of values the corresponding parameter can take during the optimization process. To execute the "arSetPars" function for all parameters at once, we provide the script "arInitValues" in the folder "D2D_extended" within our git repository. Ensure that in the script "arInitValues" the accurate path to the output from JimenaE "name_InitValues.txt" is set.
Data nodes list: "name_DataNodeNames.csv": Listing all nodes that were selected in the Experimental Data Nodes section (D2). This file is an easy way to later find and extract the corresponding data from the measurements with scripts where this list can be read in.
The script we used to read in our data can be found in the "D2D_extended" folder of the corresponding git repository.

The two main scripts generated for the D2D framework are shown in Figure sup2 and Figure sup3. For more details, please see https://github.com/Data2Dynamics/d2d/wiki/Setting%20up%20models:

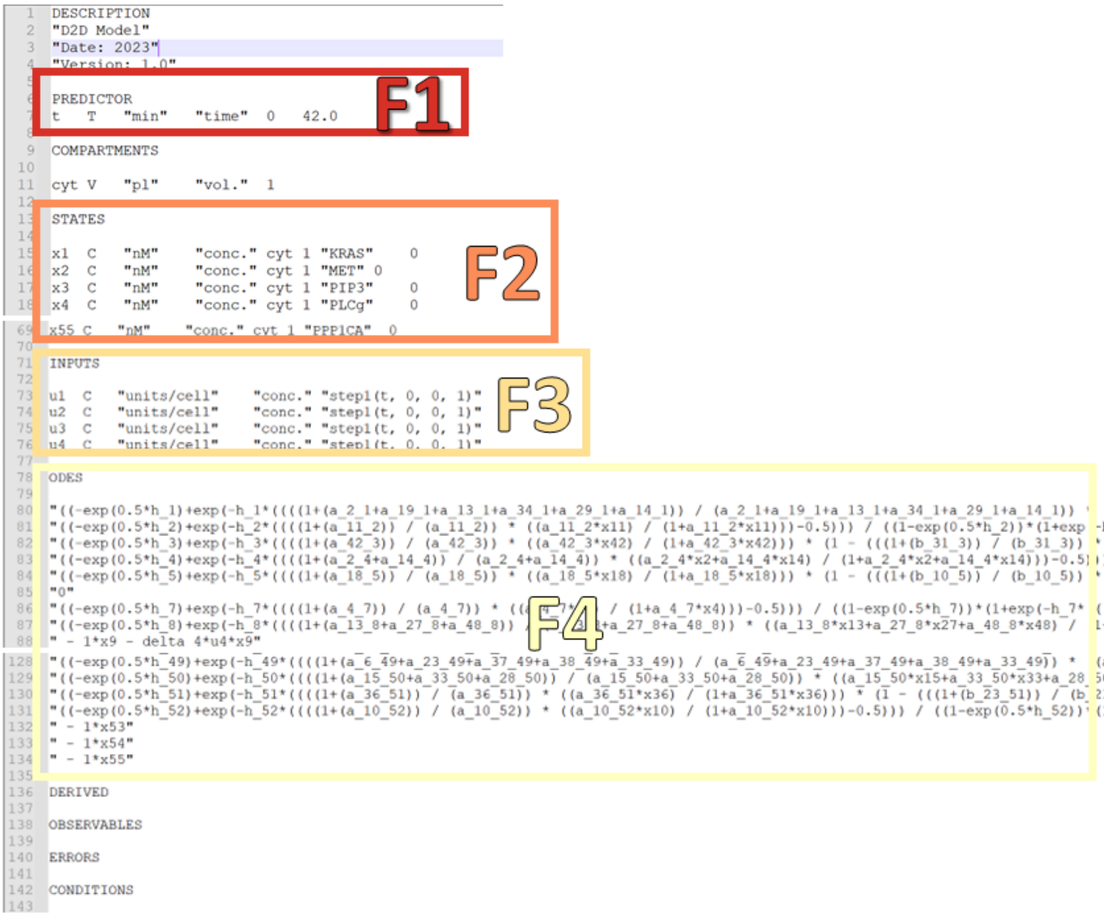

Figure sup2: The model.def file defines important model specifications for the D2D framework. A detailed description is given in the text.

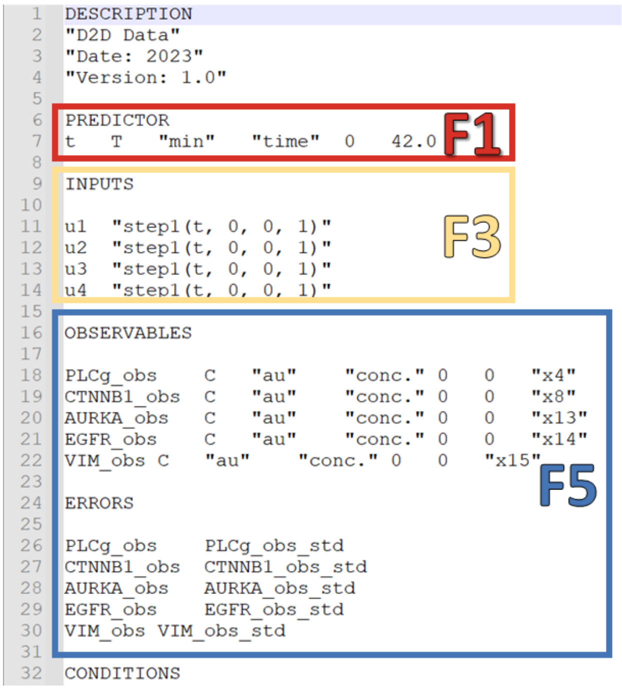

```
DESCRIPTION
"D2D Data"
"Date: 2023"
"Version: 1.0"

PREDICTOR
t    T    "min"    "time"  0    42.0

INPUTS

u1   "step1(t, 0, 0, 1)"
u2   "step1(t, 0, 0, 1)"
u3   "step1(t, 0, 0, 1)"
u4   "step1(t, 0, 0, 1)"

OBSERVABLES

PLCg_obs     C    "au"    "conc." 0    0    "x4"
CTNNB1_obs   C    "au"    "conc." 0    0    "x8"
AURKA_obs    C    "au"    "conc." 0    0    "x13"
EGFR_obs     C    "au"    "conc." 0    0    "x14"
VIM_obs C    "au"    "conc." 0    0    "x15"

ERRORS

PLCg_obs     PLCg_obs_std
CTNNB1_obs   CTNNB1_obs_std
AURKA_obs    AURKA_obs_std
EGFR_obs     EGFR_obs_std
VIM_obs VIM_obs_std

CONDITIONS
```


Figure sup3: The data.def file defines specifications about the data to which the model parameters are supposed to be fitted. A detailed description is given in the text.

F1: The PREDICTOR holds the information about the time duration of the experiment as assigned in the GUI (D5) (Fig. sup1).
F2: The STATES are the variables for the activation levels of the nodes in the network, automatically derived from the network file loaded into JimenaE. The first column provides the mathematical expression while the seventh column provides the corresponding gene name.
F3: The INPUTS define the mathematical expressions for the external stimuli. The definition in the data.def overwrites the definition in the model.def. This part can be set by the GUI (D1) (Fig. sup1).
F4: The ODES are the equations for each node (E1). They are listed in the same order as their original node counterpart was listed in the network file.
F5: The OBSERVABLES define the states for which there is measurement data as selected in the GUI (D2) (Fig. sup1). The measurement data consist of a variable representing the best value for each gene ending with "_obs" and a variable for the measurement error (standard error) ending with "_std". The data is provided with the data.csv file. We provide an example of a data.csv in folder "Example" in the provided git repository.
A data.def file is needed for each experiment. An experiment is defined as one setting of initial values of activity levels of each node of the network and one setting of external stimuli. In each run of that experiment, the initial values are the same and the external stimuli are set up the same, meaning, e.g., same intensity/concentration and time curves. For this fixed setting, the experiment is repeated several times and measurements are done after the same time after the starting point each, called t_i. For each t_i, best values and standard errors can be calculated from the corresponding measurement points. The initial values of the model are included into the fitting parameters and adapted during the best fit procedure such that the time curves of the model fit best to the time curves consisting of best values and standard errors.
In case, there are experiments that start in different initial values, we need to define different initial values such that the optimization procedure can account for that circumstance. One important further application for different initial states is the switch from one steady state to another like demonstrated in (Breitenbach et al. 2019). In such a use case, a cell is in a stable

steady state as initial state and is perturbed with the external stimuli such that it transits to a different steady state. In order to change initial values for different experiments, we can use the CONDITIONS in the data.def to overwrite the standard name of the initial value “init_geneName” as follows. Each experiment, where the system starts in the “initial value 1”, we write “init_geneName “init_geneName_1”” (without the outer quotation marks) under CONDITIONS in the corresponding data.def. Analogously, we write “init_geneName “init_geneName_2”” (without the outer quotation marks) under CONDITIONS in those data.def files where the nodes of the network initially start with the “initial values 2”. Then the optimization algorithm can take different initial states/values into account for different experiments. However, we remark, that for each repetition of such an experiment, the initial value should be the same. Otherwise, we might get high standard errors for the corresponding data points.

JimenaE explanation to set up scripts for the external stimuli framework and their description

The corresponding MATLAB scripts for the external stimuli framework are generated with the following JimenaE GUI (Fig. sup4). This GUI can be opened after loading the network file that has been used for the fitting process by clicking on the slider “Analysis” and then “D2D ExternalStimuli”.

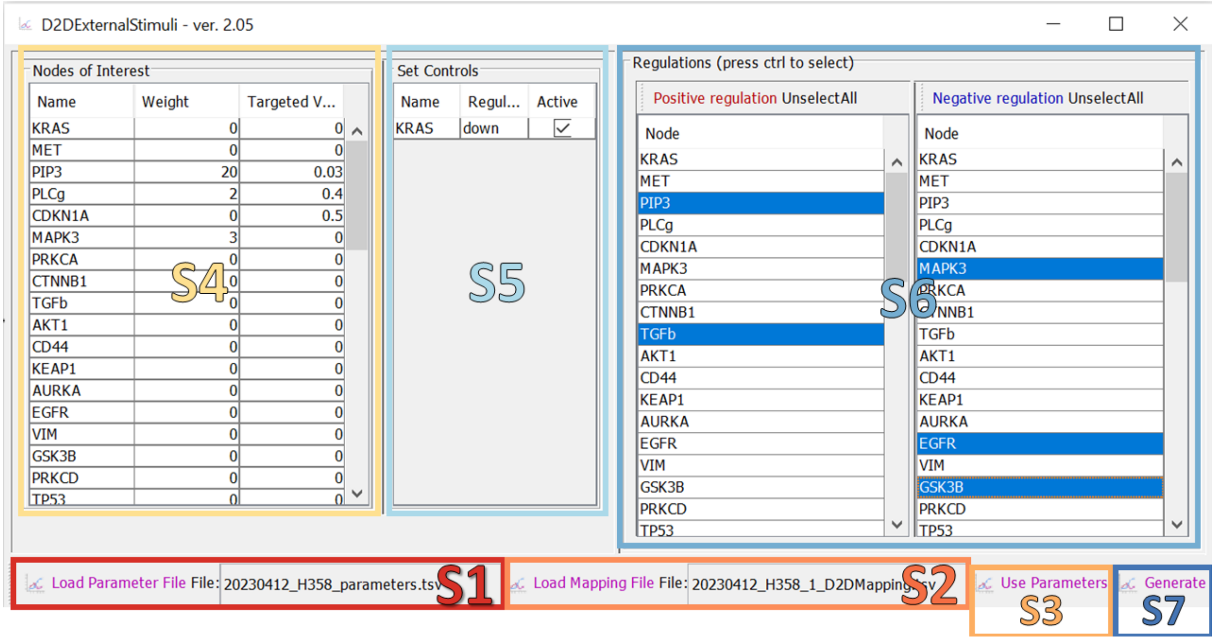

Figure sup4: JimenaE GUI to generate MATLAB scripts to apply the external stimuli framework (GUI2). A detailed description is in the text.

After opening this GUI, we have to first load two files (S1) and (S2) that have been generated by the previous GUI (Fig.sup1) and the D2D framework. The first file (S1) contains the results outputted by the D2D fitting procedure with the PEtab routine. The second file is the mapping file that is generated with the generate-button from the first GUI (Fig. sup1). Afterwards, we can specify which nodes should be a target and change their value by the influence of external stimuli (S4-S6).

S1: To enable the system to work with the parameters calculated by D2D, load the corresponding ".tsv" file. We can find this file name_parameter.tsv in the parent folder within in the D2D folder with the name PEtab.
S2: Loading in the mapping "name_D2DMapping.tsv", ensures that the parameter values from D2D are mapped correctly to their corresponding parameters in the JimenaE framework.
S3: The "Use Parameters" button allows us to set the parameters of the network to the parameter values calculated by D2D and is helpful if we plan to use other functions of JimenaE, like for example the switch analyzer (name of the method to calculate optimal external stimuli to switch between steady states, see Methods section for details) or node centrality (weights nodes with respect to their influence on the network state, see (Kaltdorf et al. 2023)), where without pushing the button, standard values are used. After pushing the buttons, just navigate to the corresponding analysis with the "Analysis"-slider.
S4: Here we can select nodes that should attain a certain target state the network is supposed to get by the influence of the external stimuli (e.g., high apoptosis value for cancer cells by the drugs or treatment of the drug targets). The target value θ_j should be between 0 and 1. The weight ϱ_j models how important it is for the user that the corresponding activity level of this node reaches the target state compared to the others. If the weight is zero, the values of the corresponding nodes are not considered in the objective accounting for deviation from the target states and is minimized subject to corresponding constraints.
S5: This list displays all nodes that were selected as Regulated Nodes in the first GUI (D2D script generation (D1)) and if they were selected as up or down regulated by an external stimulus. Unchecking a node effects the corresponding external stimulus such that it is not included into the generation of the external stimuli script.
S6: Here we can select further nodes for equipping them with external stimuli to steer the network to the desired state (defined in (S4)). The nodes selected work in the same way as described in the D2D script generator under (D1). However, if no data-fitted coupling constants (δs) are available, these additional parameters are set to 1. We remark that any other choice can be made here and is as good just by the fact that we have no data. Maybe, there is an educated guess how to set these values inspired by similar drugs. In case it turns out that results are sensitive to the concrete choice, we recommend to purposefully perform corresponding experiments to generate the data. In case of drug target identification where there is no drug available yet to influence the network accordingly, we can look at the state affected by the corresponding external stimulus and see the changes by the action of the external stimulus. Since the coupling constant and the external stimulus are a product, the optimization procedure sets the activity level of the external stimulus accordingly such that the product itself has optimal values. From the analysis and the corresponding change of the state between with and without the action of the external stimulus, although we do not have a data-determined coupling constant, we have an estimation of the effect strength a potential drug needs to have in terms of the ability to change the activity level as calculated to have the desired effect on the network.
S7: After loading the two files (S1) and (S2), defining target states (S4) and external stimuli (S5) and (S6), we can now generate the MATLAB scripts for the external stimuli framework. The button will open a window allowing us to select the location and name of the newly created file in the same way as explained in the prior GUI under point (D6). The outputted file needs to be included into a folder with auxiliary functions. These auxiliary functions are provided in our git repository under the folder "external_stimuli".
The main components of these scripts are explained in the following and depicted in Fig.sup4. The file name ends with "_main.m".

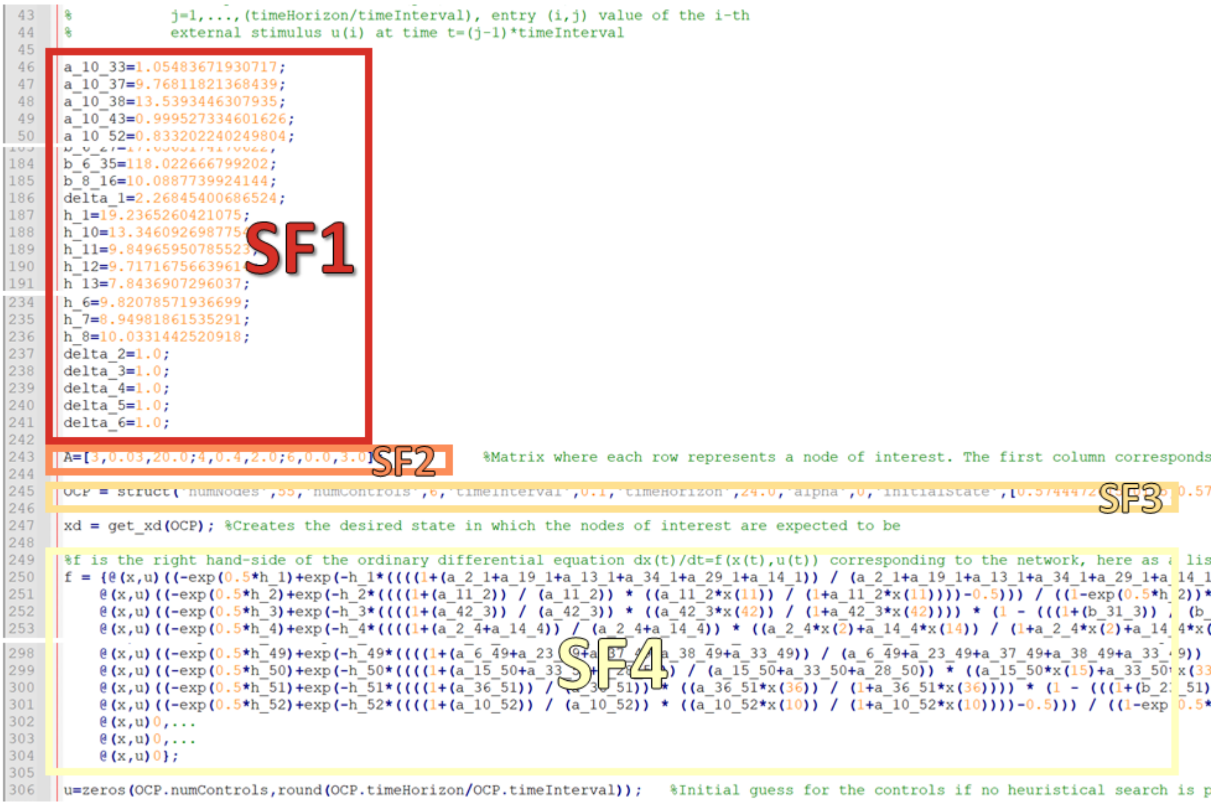

Figure sup5: MATLAB script for the external stimuli framework generated by our JimenaE pipeline. Details can be found in the text.

SF1: The list of parameters with their values calculated by D2D to fill in the variables in the equations below. Any deltas for which no fitted values are available (e.g., added as the effect of a new potential drug onto the corresponding target node) are set to 1. This value is used in the equations for both up and down regulated nodes as selected in the GUI under point (S6). Also see there for a short discussion on the choice of the values for the coupling constant in case of no data to fit them accordingly.

SF2: Here all nodes selected as nodes of interest under point (S4) will be listed as long as their set weight ϱ_j is greater than 0, as elsewise the targeted value wouldn't have any effect on the objective to be minimized anyway. For each node there are three values separated by "," while different nodes are separated by ";". The three values are the node number representing the node in the external stimuli framework, the targeted value the activity level is supposed to have, and the set weight to weight the corresponding deviation of an activity level from its desired value. The node numbering follows the same order as they were listed in the original network file and the order of the equations.

SF3: Here the number of nodes, the number of external stimuli/controls (both set automatically by JimenaE), the time delta for the discretization of the time for solving the underlying system of equations (E1) (standard 0.1, can be decreased in case of numerical instabilities solving the system of equations), the total time horizon which should coincide with predictor in the D2D script (automatically set), the parameter alpha, coincides with α introduced in the optimal control problem above, weights costs of the active external stimuli (values greater than zero) compared to nodes of interest that they are close to their target values (for details see ((Breitenbach, Lorenz, and Dandekar 2019)), and the initial states of the nodes that fit best to the data (filled automatically from the D2D results) are listed. We remark that these scripts

are for a scenario where each experiment starts in the same initial state. For a multi-initial state scenario, see next paragraph.
SF4: The equations for each node as in (E1) are listed here.

Until now, we have considered the case where the objective is to hold the network in a state by the external stimuli that is not necessarily permanent meaning if the external stimuli decay, the targeted node values go back to the initial ones.
In the following, we describe how we can use the framework to calculate optimal external stimuli to switch between to steady states meaning to transit the network from the initial state into a new state where it remains even when the external stimuli decay, see (Breitenbach et al. 2019) for details or the beginning of the Methods section.
For this purpose, we extended the original Switch Analyzer (Breitenbach et al. 2019) for the usage with D2D. To fit the network to different initial states, please see the Supplement "JimenaE GUI explanation to set up D2D scripts and their description" in the end about using the CONDITIONS.
After having found the fitting parameters, we make use of the parameters in the Switch analyzer as follows. We click in the slider "Analysis" on "D2D External stimuli" and in the GUI that opens in the bottom right corner "Use parameters" where we need to provide a path to the mapping file and the parameter file, all output from the fitting process. Now, the parameters are deposited in the JimenaE.
To generate the needed file to find efficient external stimuli to trigger the switch, we first have to search for steady states of our model in JimenaE. This function is listed in the Analysis menu under "Find Stable States". Click again into the slider "Analysis" and then "Find stable states". After the search is done, a GUI will pop up showing all found steady states and different options for how to continue. Note that not all networks have stable steady states, or even if they have, they might not be found due to limitations of a used search method. To continue, we select the "D2D Switch Analyzer" button in the lower right corner of the GUI.
A GUI is generated after selecting this option looking like Fig.sup6.

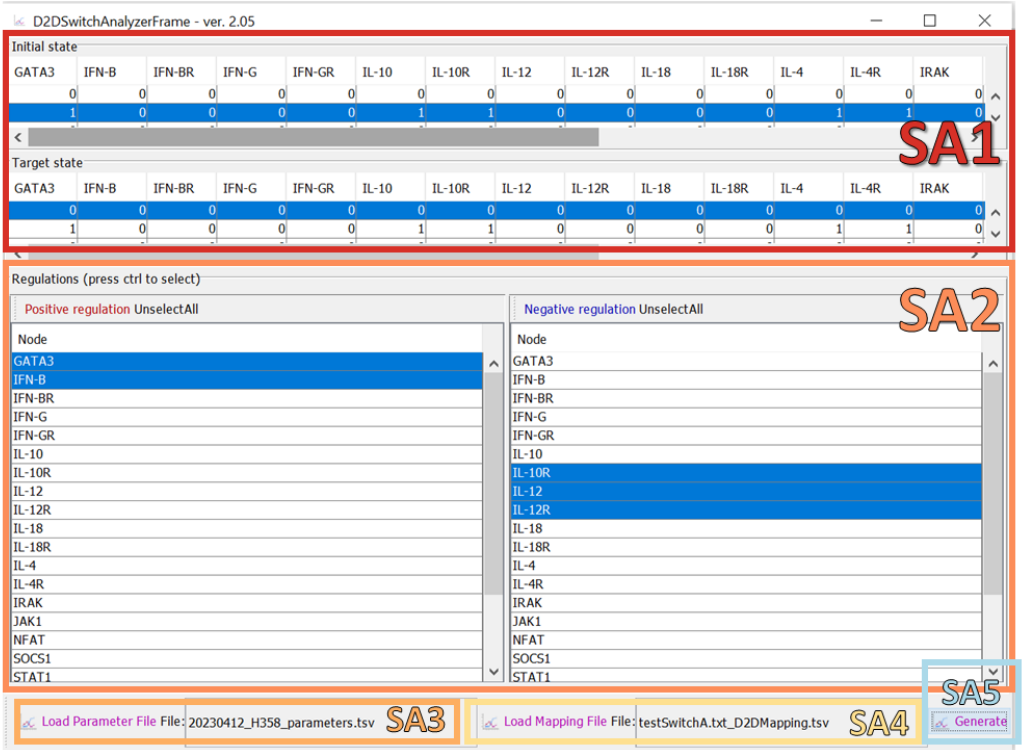

Figure sup6: GUI to generate the MATLAB script to calculate optimal external stimuli. Details are given in the text.

SA1: Here we can select any pair of states we want to analyze for their switching behavior. One initial and one target state. The external stimuli will be optimized to find the most effective combination to steer the network from the initial state to the target state.
SA2: This part allows us to select nodes we intend to perturb with the activating or inhibiting external stimuli to perform the transition of the network from the initial to the target state.
SA3: Loading in the parameters as calculated with the D2D framework. This part is the main benefit over the regular Switch Analyzer, as it allows us to include the finetuned/adapted parameters.
SA4: To ensure the values in the parameter file are mapped to their corresponding parameters in the equations, we additionally have to load the mapping file, which was created with the button under (D6). We find that file at the same location where all other files created by the D2D GUI triggered by (D6) are located.
SA5: This button allows us to generate the optimal external stimuli MATLAB script needed for the analysis of switching a network between two steady states. Its generation is carried out in the same way as the file creation in the other described GUIs.
The name of the files created with that button end with "_main.m". In Fig.sup7, we explain the main parts.

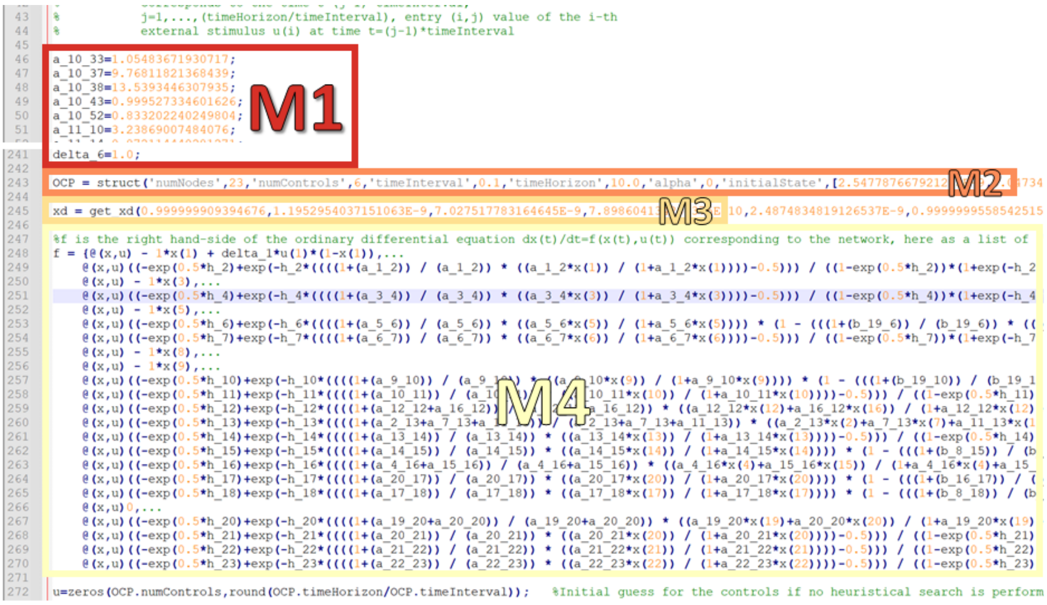

Figure sup7: MATLAB script Main.m generated with JimenaE for calculating optimal external stimuli for switching between two steady states.

M1: The parameters as calculated by D2D, with any new regulations (SA2) added as corresponding deltas.
M2: The state selected as the initial one (SA1) where the other parameters have the same meaning as in the other external stimuli MATLAB script.
M3: The state selected as targeted state (SA1).
M4: The equations determining the network behavior for each node.

Fitting tutorial

In this section, we present how to adapt the gene interaction as depicted in Fig.sup8(A) to the single cell data. There are 20 nodes shown in the topology and display the pathway regulation in a typical cancer (Fig.sup8(A)). The optimal state to which the D2D best fitting procedure converges shows oscillations in almost all curves. We see that the oscillating curves go through the data points, however, we favor a solution that has constant expression values which might fit better the real situation. We assume that the oscillations rather come from the fact that the number of our measurement points are too few compared to the number of free parameters that we fit. Together with the fact of too few measurement points, we would like to remark that we cannot use the output of the Chi2Test function of D2D to evaluate if the model fits the data based on a level of significance, meaning how likely it is that deviations between data and model are rather explainable with noise instead of systematic model errors. The reason is that the corresponding p-value is not defined due to a too little number of measurement points compared to the number of parameters that are fitted. For details about the rationale about utilizing the chi-square test for model fitting, please see the Methods section. For more details about the background of the chi-square test, please see (Breitenbach et al. 2022). We explain in the Discussion how a procedure can look like to increase measurement points purposefully in accordance with the presented framework. We remark that in terms of an effective data evaluation the measurement of data points needs to be considered with the research question and the methods used for analysis. One of our aims of this work is to lower the effort for the application of data fitting and model analysis regarding efficient drug targets to facilitate data

generation and its evaluation at the same time. In the presented work, we limit ourselves to a visual evaluation for fitting which showcases just as well the application of the total framework and at the same time demonstrates that our method can be used in the case of a sparse data foundation. Under section “Enlarged image of fitting process” all images of the fitting results are displayed solely to view them at a larger size.

To improve the fitting of the model with the data, we assume that the oscillation could be caused by the missing inhibiting input of the node FBXW7 (Fig.sup8(B)) since it seems too highly expressed causing a too strong inhibition of AURKA and an inhibition could damp the oscillations.

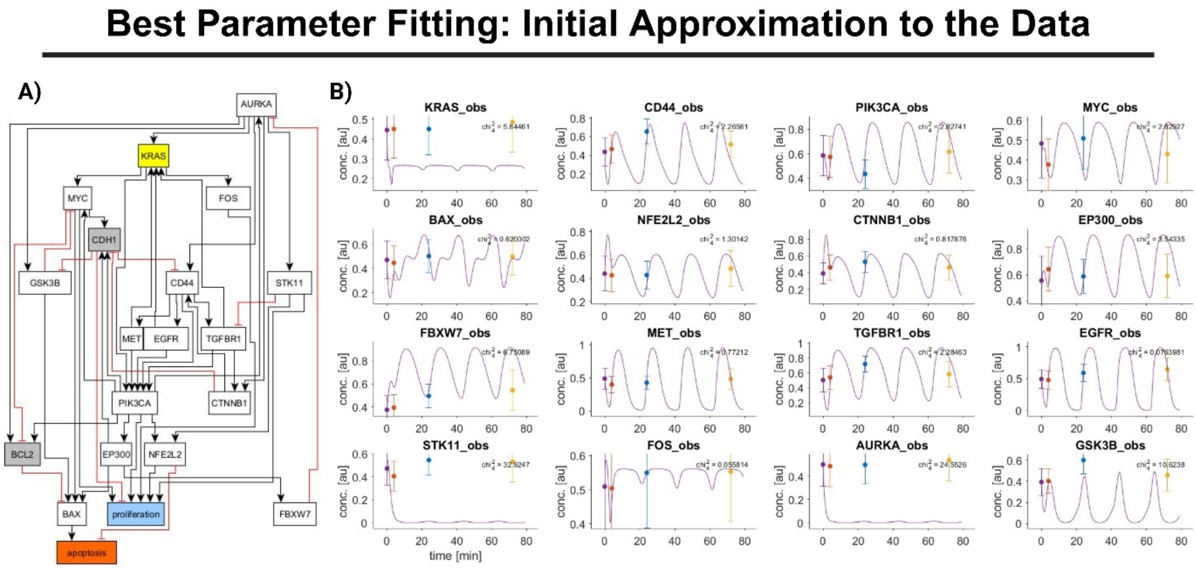

Figure sup.8: Best parameter Fitting: Initial Approximation to the data. A) A topology of a protein-protein interaction of the H358 cell line (modified after (Peindl et al. 2022)). Based on data, a yellow node symbolizes the node under treatment conditions (KRAS-Inhibitor). The primary functional endpoints within this network are represented by apoptosis nodes in red and proliferation nodes in blue, highlighting important cellular processes. Apoptosis (red node) and proliferation (blue node) serve as output nodes. Grey nodes are without any data. **B)** Fitting results of 16 measured genes (18 nodes serve as regulation nodes, two as output). For each gene, four data points are visualized in the graph depending on time points (0h(purple), 4h (red), 24h (blue), and 72h (yellow)). In the protein-protein interaction network, each gene is accompanied by a chi-square value, total Chi2 = 97.8907 with 64 data points and 91 free parameters. Created with BioRender.com.

To overcome this problem of oscillations, the model was modified by extending an inhibitory fictive node with a constant expression value selected in the GUI (Fig.sup1 (D3)) named F-Box And WD Repeat Domain Containing 7 (FBXW7) (Fig.sup9(A); green). The idea of a fictive node is that we can test the effect of a potential gene with its inhibitory effect (in general with the required property of activating or inhibiting) if it pushes the model in our favor and if yes, one can search for a corresponding gene with similar expression values. In other words, we can postulate the action of another gene, focusing on a targeted search for such a gene and as we see later in our demonstration, we found a gene with the postulated influence on the network. This is a further example, how our proposed pipeline can accelerate research by giving guided hints for improvements from a data-driven evaluation.

Technically, we change the initial value in the updated_arSet.tsv file, explicitly the arSetPars ('init_x21','initial value',1,0,0.0,1.0), which is the function to set the initial value for the fictive

node for the parameter optimization method, to 0.3 ('init_x21','initial value',0.3,0,0.0,1.0). Such variations in the initial guess starting from 0.3 instead of 1.0 can cause the gradient method to converge to a local optimum with better fitting timer curves. We remark that finding well working initial guesses is sometimes trial and error work. However, with our framework, we can narrow down this trial-and-error work to particular single cases, e.g., a single fictive node that acts on a node that has a high deviation from its data measured with regard to its contribution to the chi-square value. This value change provides an improvement of the model (Fig.sup9(B)) regarding fitting to the data. While the best-fit plots are generally in good agreement with the data, the plot of Aurora Kinase A (AURKA) stands out with its deviation from the data, as evidenced by a high chi-square value of 7.25452 (Fig.sup(B); red box), compared to the genes as one of the major contributions to the total chi-square value of the model. This discrepancy might result from the weak activation of AURKA.

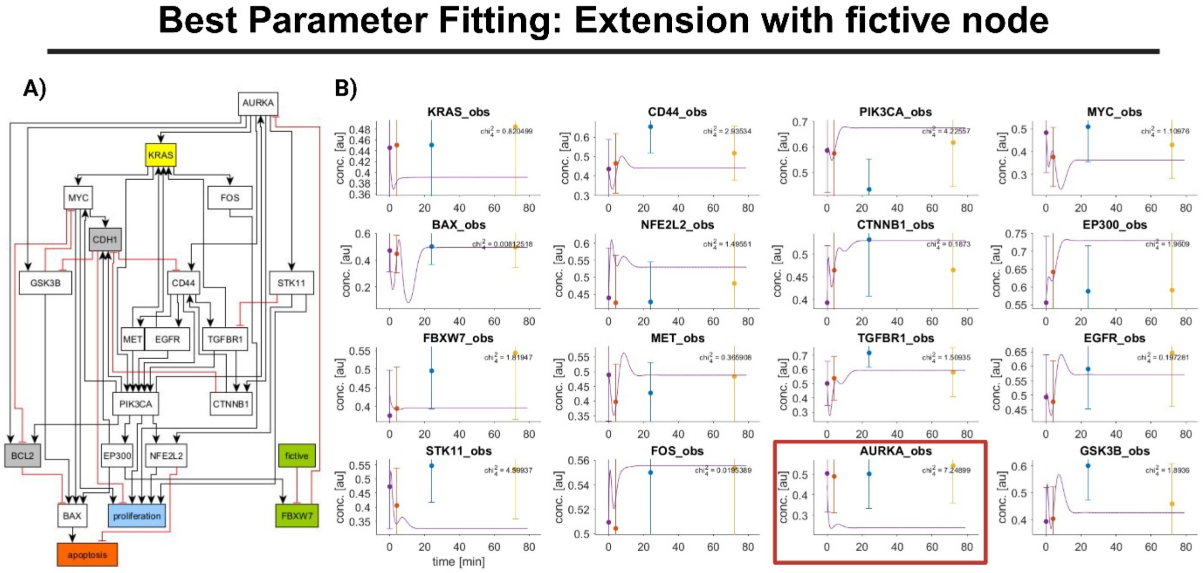

Figure sup9: Best Parameter Fitting: Extension with a fictive node. A) The protein-protein interaction network for the H358 cell line (modified from (Peindl et al. 2022)) features an extension with a fictive node highlighted in green. This fictive node exerts an inhibitory influence on FBXW7 (green) within the network. The node highlighted in yellow, represents the node KRAS under treatment conditions with a KRAS inhibitor. Major functional endpoints are represented by apoptosis (red) and proliferation (blue) nodes. Nodes for which no data is available are shown in gray. B) This figure illustrates the fitting results for 16 measured genes within the context of a protein-protein interaction network. In the network, 18 nodes serve as regulatory nodes, while two nodes function as output nodes. Each of the 16 genes is depicted by four data points, corresponding to distinct time points: 0 hours (purple), 4 hours (red), 24 hours (blue), and 72 hours (yellow). Additionally, accompanying each gene in the network, a chi-square value is provided to gauge the goodness of fit for the respective gene's expression data, total Chi2 = 30.4133 (64 data points, 91 free parameters). The red box symbolizes the node AURKA, which has the highest chi-square value (Chi2 = 7.25452). Initial value for x21= 0.3. Created with BioRender.com.

To improve the fitting of AURKA, a fictive node is inserted to strengthen the activation of AURKA and all best-fit parameters from the previous run were taken to improve the fit to the data (Fig.sup10(A); orange; (B)). The activity of AURKA exhibited a negligible change, resulting in a slight improvement in the chi-square value, which now stands at 18,947. This outcome occurred after adapting the optimal fit parameters from the preceding run and initializing the initial value of the fictive node x22 (fictive2) with 1 and of the fictive node x21 (fictive) with 0.16. These fictive nodes are modeled as nodes with a constant activity level. In Fig.sup10, we

see the node “fictive” in green and the node fictive2 in orange associated with AURKA and FBXW7. These changes are made in the file UpdateSetPars.txt. The “arBestFit_SetPars” does not have to be performed repeatedly (can be commented with a % in the advanced_script (Fig. 3) and only “arUpdateSetPars” with the changed values is used. The total chi-square value of 68.9107 is increased mainly by the Hepatocyte Growth Factor Receptor (MET) node, which has a chi-square value of 40.5377 (Fig.sup10 (B); red box).

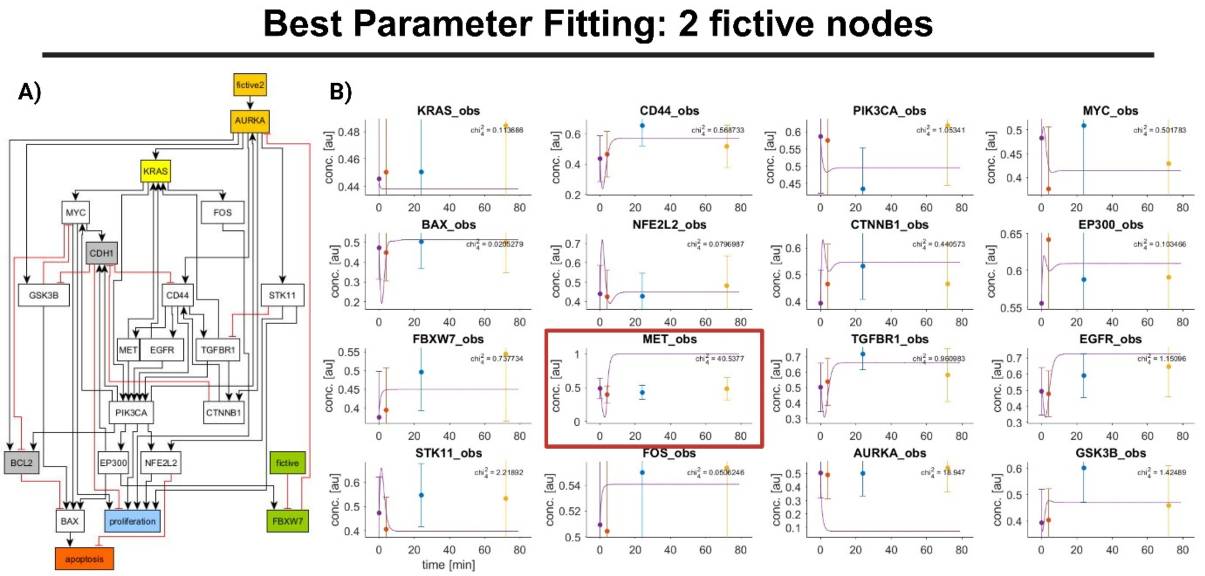

Figure sup10: Best Parameter Fitting: 2 fictive nodes. A) The protein-protein interaction network for the H358 cell line (after (Peindl et al. 2022)) was extended by a second fictive node, both marked in green and orange. These fictive nodes play different roles within the network and postulate the action of genes that we later identify in our gene set. The green node exerts an inhibitory influence on FBXW7, while the orange node serves as an activator for AURKA. In addition, a node highlighted in yellow indicates KRAS, which is under treatment conditions with a KRAS inhibitor based on the data. The primary functional endpoints within this network are represented by apoptosis nodes in red and proliferation nodes in blue, highlighting important cellular processes. Nodes for which no data is available are shown in gray. B) The fitting results for 16 measured genes within the context of a protein-protein interaction network are visualized. In this network, there are 18 nodes serving as regulatory nodes, and two nodes specifically functioning as output nodes. Each of the 16 genes is represented by four data points, corresponding to distinct time points: 0 hours (purple), 4 hours (red), 24 hours (blue), and 72 hours (yellow). In addition to each gene’s representation in the network, a chi-square value is provided to assess the goodness of fit for the respective gene’s expression data. The total chi-square value is calculated as 68.9107, considering 64 data points and 95 free parameters. Notably, the node MET is highlighted with a red box due to its association with the highest chi-square value (Chi2=40.5377). For the optimization run, the start value for both fictive nodes are set up as arSetPars('init_x22',1.0,1,0,0.0,1.0) and ('init_x21‘,0.16,1,0,0.0,1.0). Created with BioRender.com.

To improve the fit to the data, an additional third node with a constant expression value, which inhibits MET, is added (Fig.sup11 (A)) and all previous best fit parameters are taken from the calculation before. After fitting, the total chi-square value is 10.7794. This fitting is performed by updating the initial values for x22 (fictive2) and x23 (fictive3) to 0.5 for both in the “arUpdateSetPars” function. This result of a low total chi-square value in a sum over all 16 nodes compared to our first version of the topology indicates a good fit to the data. Also visually, the curves fit well to the data points within their standard errors. Our next step is to find representative biologically corresponding nodes for the fictive nodes in the model to make our model closer to the modeled system.

Best Parameter Fitting: 3 fictive nodes

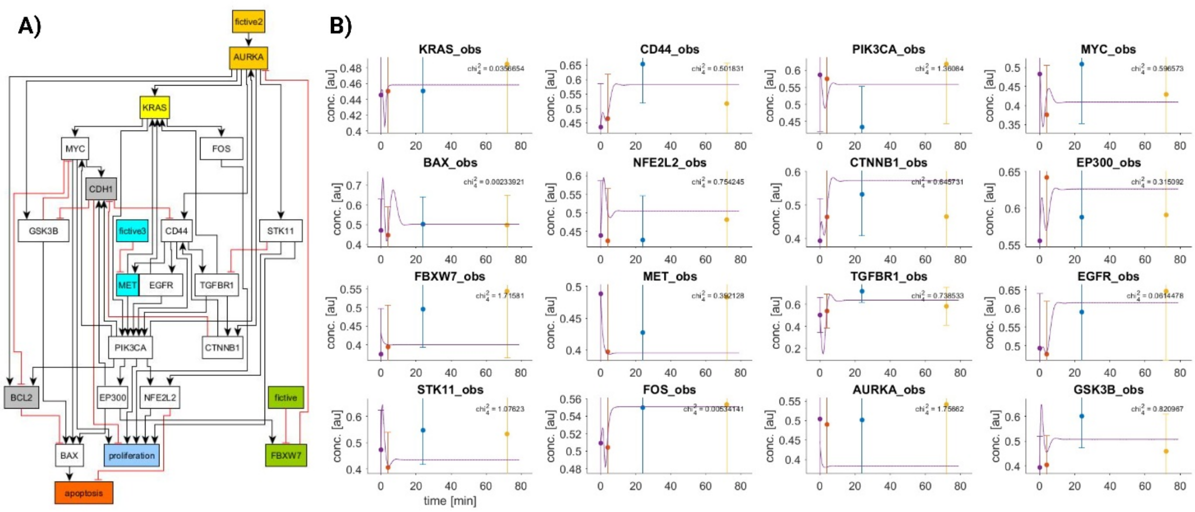

Figure sup11: Best Parameter Fitting: 3 fictive nodes. A) An extension of the protein-protein interaction network (modified after (Peindl et al. 2022)) for cell line H358 by a third fictive node (turquoise). The turquoise node exerts a suppressive effect on MET, which is also shown in turquoise and represents a tyrosine kinase receptor. Nodes for which no data is available are shown in gray, whereas the yellow node corresponds to the KRAS node under certain treatment conditions with a KRAS inhibitor. The major output nodes are color-coded, with red representing apoptosis and blue representing proliferation. B) Fitting results for 16 measured genes within a protein-protein interaction network are displayed. This network consists of 18 regulatory nodes and two output nodes. Each gene is represented by four data points at varying time intervals: 0 hours (purple), 4 hours (red), 24 hours (blue), and 72 hours (yellow). Chi-square values are provided for each gene to assess the goodness of fit for their expression data. The total chi-square value is 10.7794, considering 64 data points and 95 free parameters. The initial values in the optimization routine for x21, x22, and x23 are 0.16, 0.5, and 0.5, respectively and are adapted during the optimization run such that the activity levels of the nodes fit the data best. Created with BioRender.com.

The search for genes to replace the fictive nodes leads to 3 genes. For two of these nodes, Microtubule Nucleation Factor (TPX2; orange) and Dual Specificity Tyrosine Phosphorylation Regulated Kinase 2 (DYRK2; green), measurements were performed and included as nodes with a constant expression level into the fitting processes, please see Fig.sup1 (D3) how to set nodes to a constant expression level. The fictive node on MET is replaced by TP53 as an inhibitory influence on MET, reported in (Zhou et al. 2023). However, we do not have measurement data to examine if the fitted constant expression value matches the measurements within their standard errors, as we see it for the other two genes. The optimization method then only optimizes the initial value, which the corresponding node constantly takes for the whole time, such that the chi-square value is minimized. The node for inhibition of MET, the Tumor Protein P53 (TP53) gene, serves here as a potential node that can be verified in further experiments with corresponding data. We see that how our proposed framework can guide data acquisition purposefully to genes that seem meaningful to improve and extend a model targeted. In a future study, we could conduct a more in-depth investigation to identify genes that potentially exhibit suitable interactions. In any case, the fictive node provides the information that an inhibiting effect from some gene is probably missing but could be postulated based on our currently available data. The fitting process with a total chi-square value of 19.951 is slightly increased by the new data input of TPX2 and DYRK2. The main reason for this increasement is that DYRK2 shows a higher activation in the data than needed in the model. The result is shown in Fig.4.

Tutorial for finding most efficient intervention points

We showcase the application of the external stimuli part of JimenaE for identifying drug targets based on real data from a H358 cell line. The best fitting model is used, which is given in our git repository in the “Example” folder in the PEtab, imported as yED graph (yEd 2019) (the topology file is available as H358_topology in our git repository in the “Example” folder), and analyzed with "D2D ExternalStimuli" (Fig.sup4). The following configurations have been made: The nodes of interest, namely apoptosis and proliferation, were established as target nodes with desired values. Both were assigned a weight of 1, with a target value of 1 for apoptosis and 0 for proliferation. In this context, the primary focus is on apoptosis to combat cancer, but also proliferation should still be downregulated. Possible drug-target nodes are selected taking into consideration whether they should receive a positive (activating) or negative (inhibitory) regulation (Fig.sup4). Further, a parameter file from the best fit (in the folder PEtab → parameter) and mapping file (output JimenaE → generated via 1. Step (Fig. 1)) are selected, both given in our git repository. After loading the files, the previous external stimuli are automatically set up in the “Set controls” (Fig.sup4). Afterward, a MATLAB script is generated using the "generate" function (Fig.sup4) and given a specific name. The file is saved in the directory where it will be executed alongside the external stimuli helper functions (folder “external_stimuli” in the git repository).

For starting the analysis, the generated script is opened in MATLAB. The execution of this script in MATLAB requires the “Symbolic Math Toolbox”. The toolbox "Parallel Computing Toolbox" package is optional. If the “Parallel Computing Toolbox” is not available, just replace “parfor” by “for” in the corresponding scripts switching off the parallel computation of the corresponding for-loops.

To calculate external stimuli, at least one of the flags “combi_method” or “local_optimization_method” must be set to 1. For expedited calculations, the tolerance parameter in line 17 of the JimenaE output file name_main.m has been adjusted from tol2=10^-14 to tol2=10^-7. For our use case, this accuracy turned out sufficient meaning that a higher accuracy did not influence if an external stimulus is constant zero or non-zero. Before the script is executed, the alpha parameter must be configured. The parameter alpha is used to weigh the initiation of an external stimulus to a complete inactivity of the corresponding external stimulus. The external stimulus is sometimes called control as a synonym, which is used in, e.g., Fig.sup4 or Fig.sup5. Essentially, a higher alpha value means that the external stimulus must be more effective in terms of steering the nodes of interest to the desired state not to be set to constant zero. The higher alpha, the more effective the contributions of the external stimulus have to be such that it is non-zero. Consequently, increasing alpha for each run solving the optimal control problem leaves only the most effective external stimuli non-zero achieving a high apoptosis and a low proliferation. We choose alpha equal to 0.1 as a starting point. As a result, two plots are shown, Fig.sup12 and Fig.sup13. The first diagram depicts the observed external stimuli that are most effective in achieving the desired state, which involves downregulating proliferation and upregulating apoptosis. For the calculation, 14 external stimuli were used affecting genes such that they are down-regulated (KRAS, MYC, NFE2L2, EP300, MET, TGFBR1, EGFR, STK11, AURKA, CD44, PIK3CA, FOS) and up-regulated (CDH1, GSK3B). The inhibiting external stimulus on KRAS results from the data since the data was generated by administrating the KRAS inhibitor. Consequently, the corresponding external stimulus needed to be set active in the fitting process of the model and is automatically set in JimenaE (see Fig.sup1(D1) and its explanations for details). The exact time curves of the external stimuli influencing the corresponding nodes are not essential for our study but the information which external stimuli are

set to constant zero since they are not effective enough compared to the non-zero ones. However, for a model fitted to experimental data, our presented framework can be used with the exact time curves of the external stimuli, which could mean a drug application in terms of amount or dosage over time. The second plot is a time-dependent graph showing the output of the "nodes of interest" (Fig.sup12(B)). The nodes of interest are states supposed to have a certain value that deviates from the scenario where no external stimulus is active. By the action of the external stimuli, we obtain a low proliferation and a high apoptosis as desired.

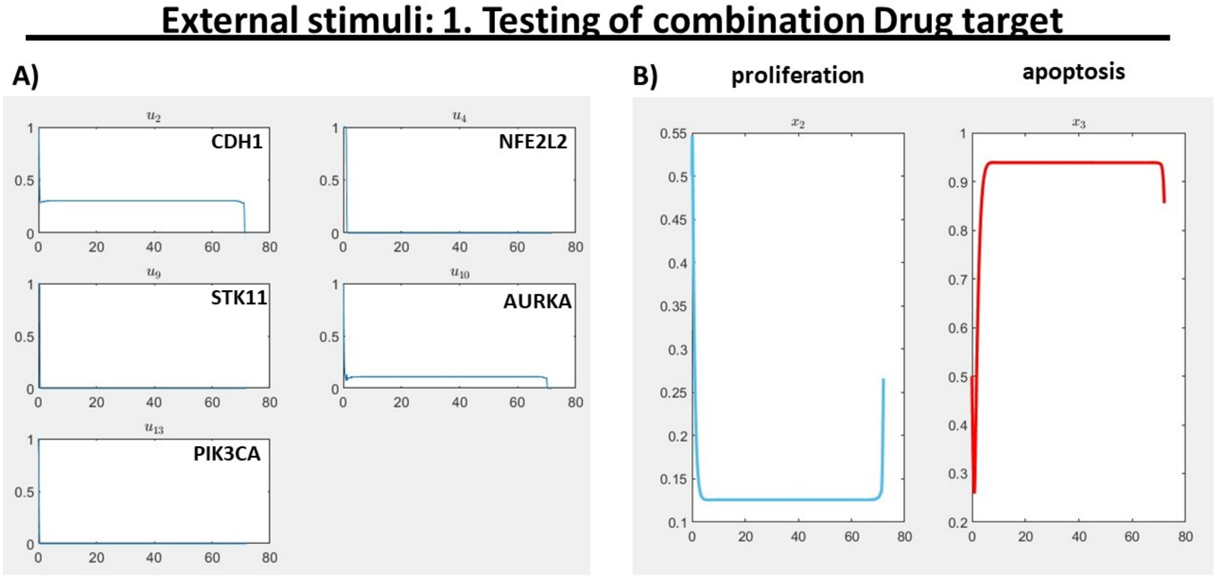

Figure sup12: External stimuli first run. To identify the impact of external stimuli, we set the alpha value to 0.1 and tested 14 external stimuli that down-regulated genes (KRAS, MYC, NFE2L2, EP300, MET, TGFBR1, EGFR, STK11, AURKA, CD44, PIK3CA, FOS) and up-regulated genes (CDH1, GSK3B). A) The plots show the activity level of the corresponding drugs influencing CDH1, NFE2L2, STK11, AURKA, and PIK3CA. B) The outcomes of this study regarding therapeutic efficacy show a downregulation in proliferation and an increase in apoptotic effects.

By increasing the alpha parameter, it becomes possible to identify more effective drug targets regarding influencing our nodes of interest and to reduce the number of non-zero external stimuli. It is essential to take the changes of the nodes of interest into account caused by the drug target selection to ensure that the chances of the external stimuli regarding the nodes of interest are not minor due to a too high alpha. A high alpha hinders an extensive use of external stimuli because their action cannot provide a corresponding benefit regarding pushing the nodes of interest to the desired values. If alpha is too high, all external stimuli are eventually constantly zero. It is always necessary to consider both plots, e.g., Fig.5 and Fig.sup13. The values of the target nodes also allow a quantification of the effect of the active external stimuli. Consequently, we can see, for example, if there are non-linear effects in terms of the number of external stimuli meaning that a second active external stimulus can bring much more effect regarding the desired nodes being close to their desired values compared to just a single drug. Increasing alpha results into the best combination of drug target nodes according to our data and model presented (Fig.6).

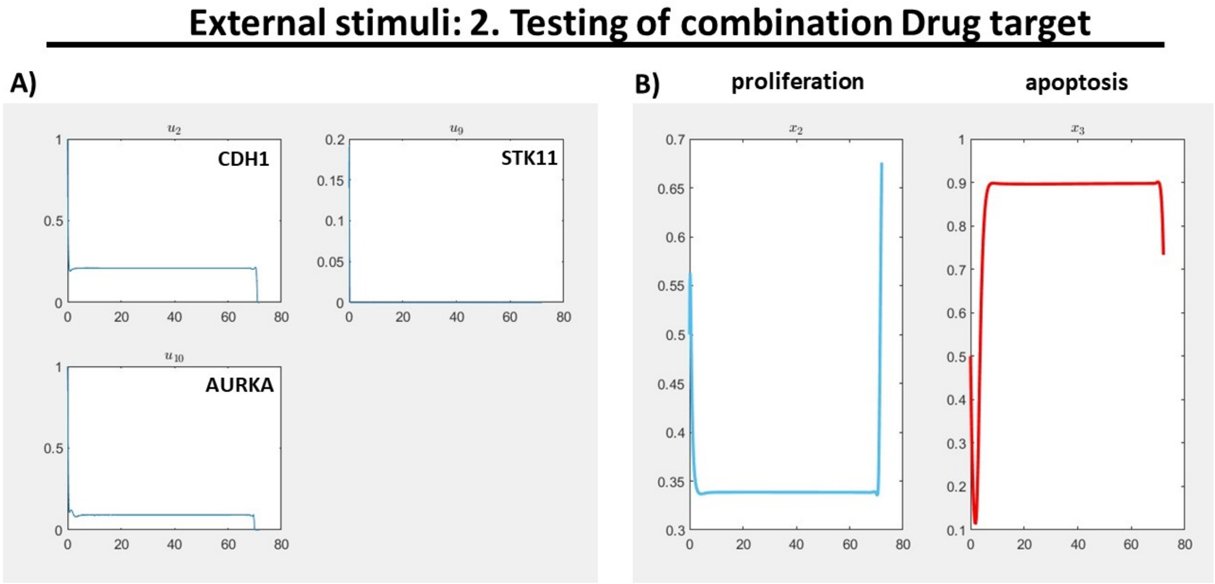

Figure sup13: External stimuli second run. To investigate the influence of external stimuli, we established an alpha value of 0.5 and conducted experiments on 14 external stimuli. These external stimuli encompassed genes that were either down-regulated (e.g., KRAS, MYC, NFE2L2, EP300, MET, TGFBR1, EGFR, STK11, AURKA, CD44, PIK3CA, FOS) or up-regulated (such as CDH1 and GSK3B). A) The findings are visually depicted in a plot, highlighting specific drug-regulated nodes namely CDH1, STK11, and AURKA. B) In terms of the study's outcomes related to therapeutic efficacy, there is an observed decrease in cell proliferation and a heightened apoptotic effect.

Enlarged Images of fitting process

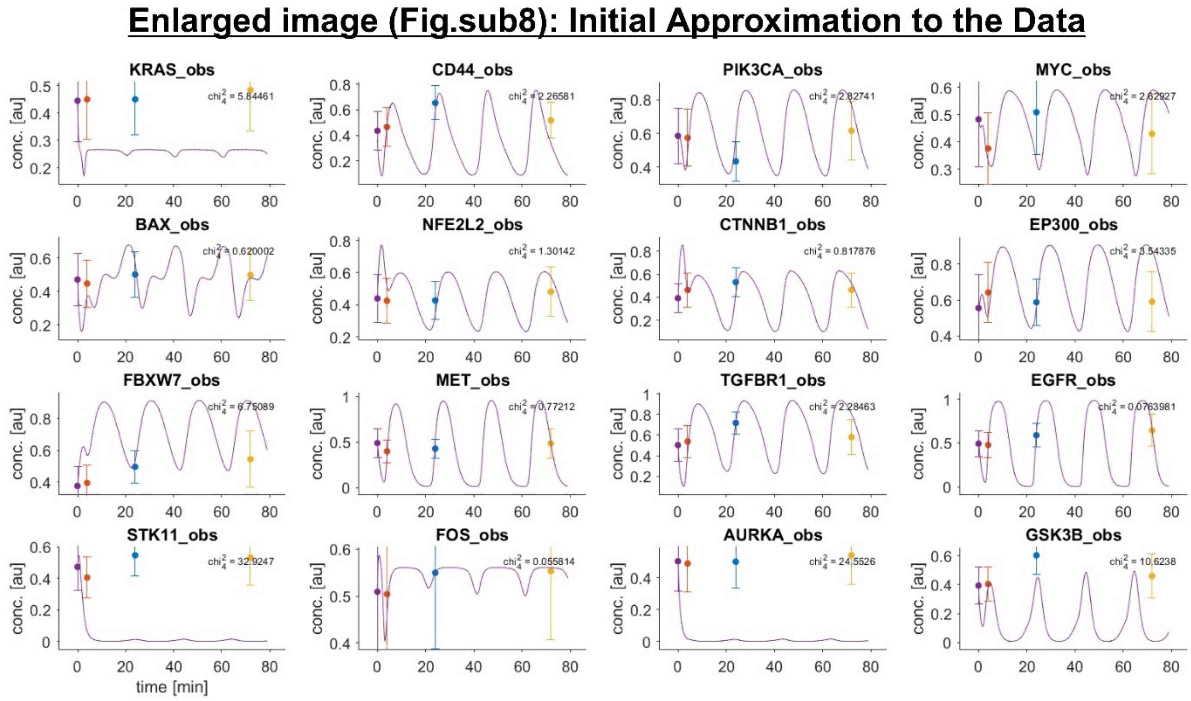

Figure sup14: Enlarged view of Fig. sup8. Created with BioRender.com.

Enlarged image (Fig.sub9): Extension with fictive node

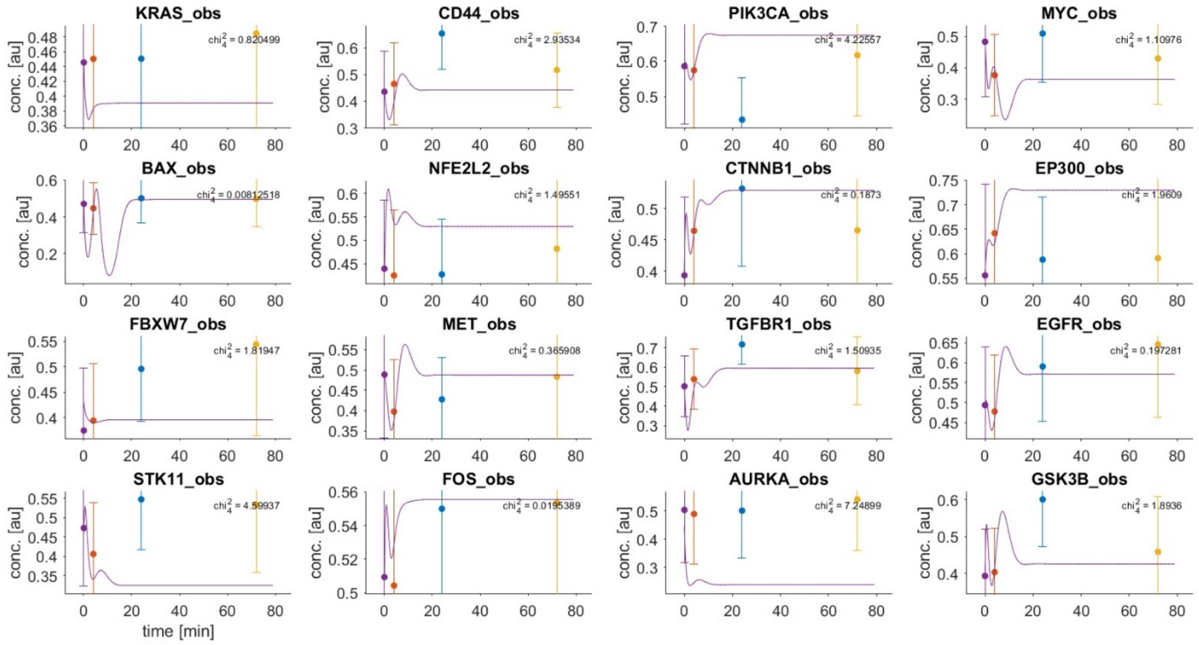

Figure sup15: Enlarged view of Fig. sup9. Created with BioRender.com.

Enlarged image (Fig.sub10): 2 fictive nodes

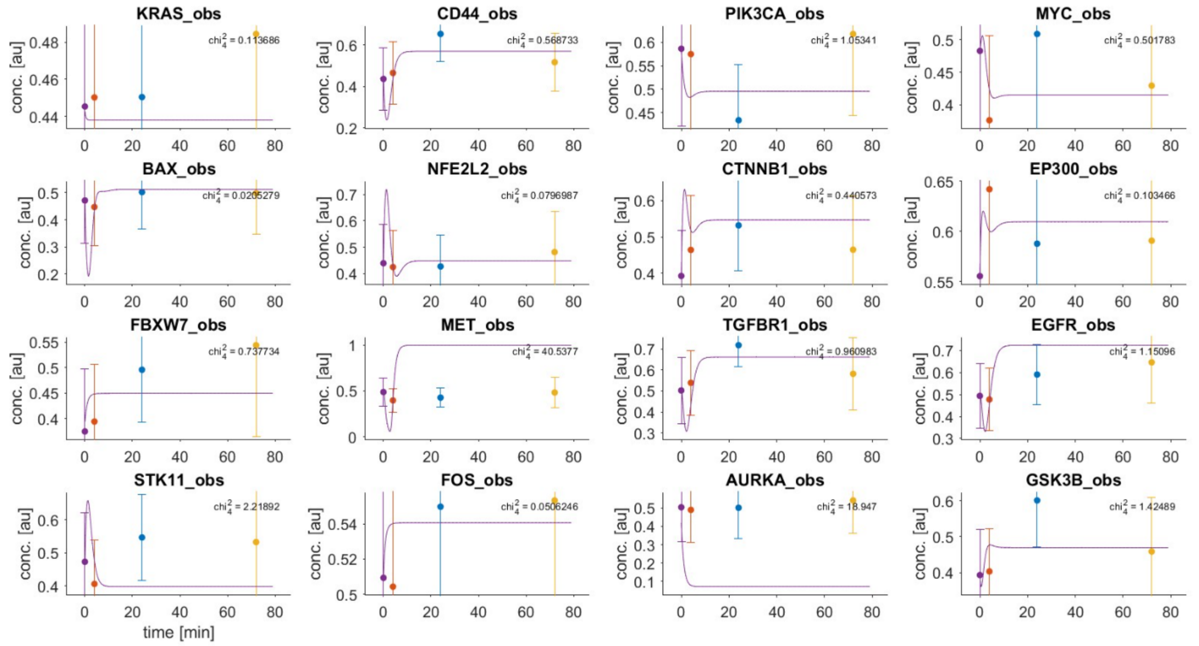

Figure sup16: Enlarged view of Fig. sup10. Created with BioRender.com.

Enlarged image (Fig.sub11): 3 fictive nodes

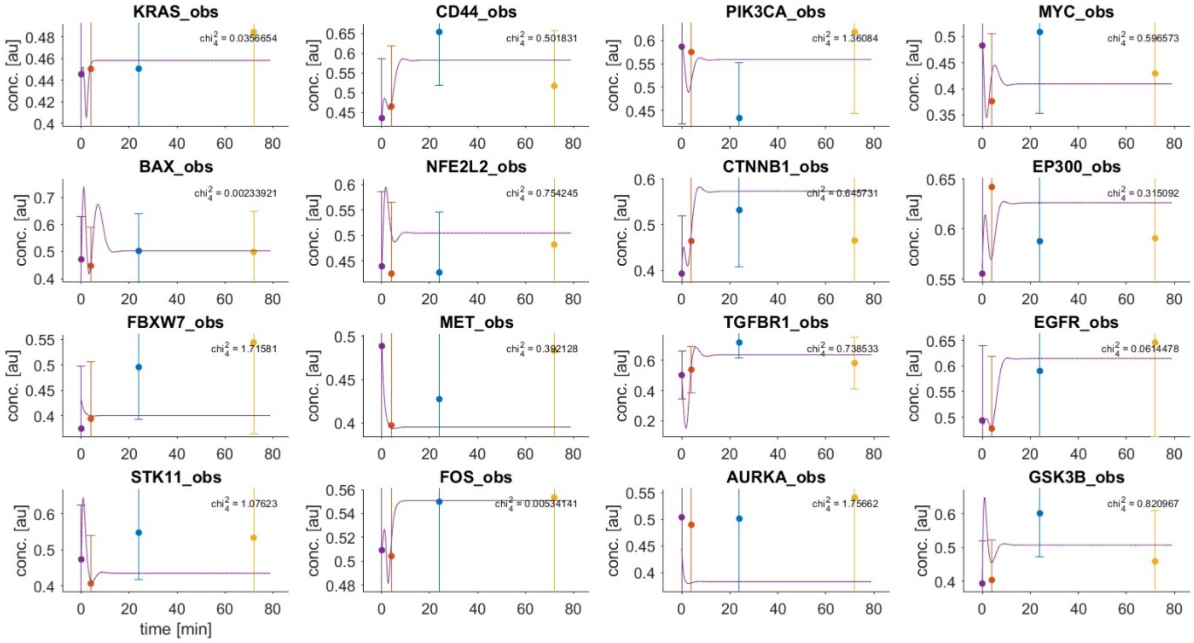

Figure sup17: Enlarged view of Fig. sup11. Created with BioRender.com.

Enlarged image (Fig.6): New nodes

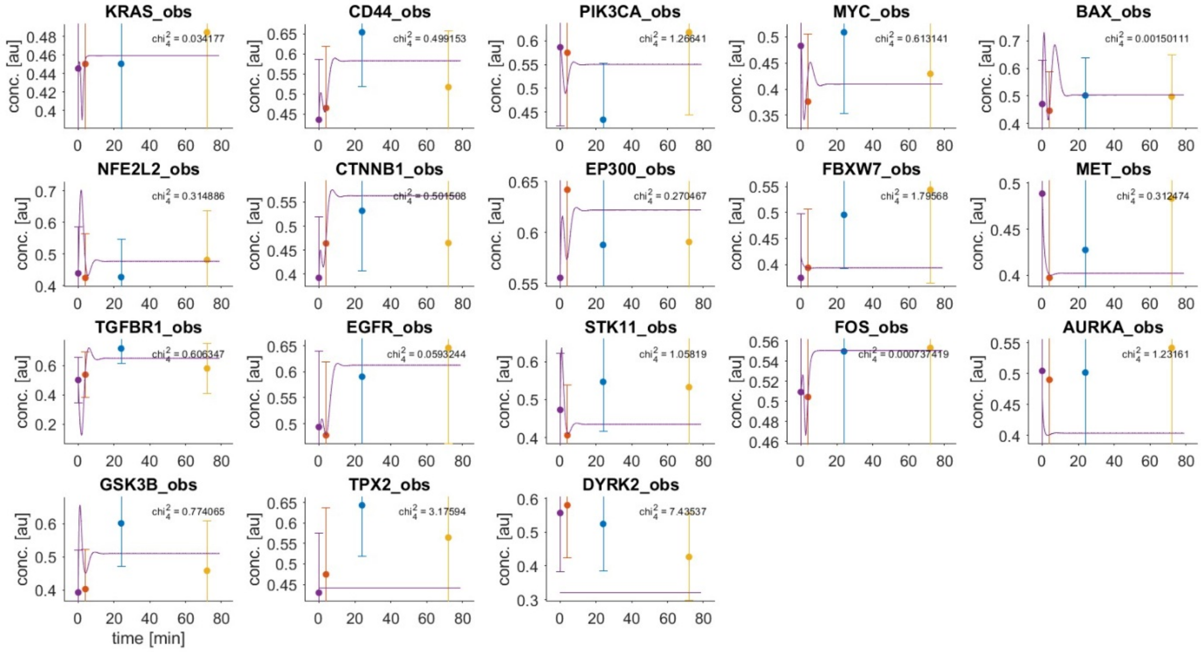

Figure sup18: Enlarged view of Fig. sup12. Created with BioRender.com.
